# China's greenhouse gas budget during 2000–2023

**DOI:** 10.1093/nsr/nwaf069

**Published:** 2025-02-22

**Authors:** Wenping Yuan, Minqi Liang, Yuanyi Gao, Ling Huang, Li Dan, Hongtao Duan, Songbai Hong, Fei Jiang, Weimin Ju, Tingting Li, Ziyang Lou, Shilong Luan, Xiao Lu, Zhangcai Qin, Lishan Ran, Lulu Shen, Fei Teng, Xiangjun Tian, Yilong Wang, Jing Wei, Jiangzhou Xia, Xiaosheng Xia, Lijun Yu, Xu Yue, Haicheng Zhang, Wen Zhang, Yuzhong Zhang, Xu Zhao, Qiuan Zhu, Shilong Piao, Xuhui Wang

**Affiliations:** Institute of Carbon Neutrality, Sino-French Institute for Earth System Science, College of Urban and Environmental Sciences, Peking University, Beijing 100871, China; School of Atmospheric Sciences, Guangdong Province Data Center of Terrestrial and Marine Ecosystems Carbon Cycle, Sun Yat-sen University, Zhuhai 519082, China; Institute of Carbon Neutrality, Sino-French Institute for Earth System Science, College of Urban and Environmental Sciences, Peking University, Beijing 100871, China; Institute of Carbon Neutrality, Sino-French Institute for Earth System Science, College of Urban and Environmental Sciences, Peking University, Beijing 100871, China; Key Laboratory of Regional Climate-Environment for Temperate East Asia, Institute of Atmospheric Physics, Chinese Academy of Sciences, Beijing 100029, China; Key Laboratory of Lake and Watershed Science for Water Security, Nanjing Institute of Geography and Limnology, Chinese Academy of Sciences, Nanjing 211135, China; State Key Laboratory of Lake Science and Environment, Nanjing Institute of Geography and Limnology, Chinese Academy of Sciences, Nanjing 211135, China; School of Urban Planning and Design, Shenzhen Graduate School, Peking University, Shenzhen 518055, China; Frontiers Science Center for Critical Earth Material Cycling, Nanjing University, Nanjing 210023, China; Jiangsu Provincial Key Laboratory of Geographic Information Science and Technology, International Institute for Earth System Science, Nanjing University, Nanjing 210023, China; Frontiers Science Center for Critical Earth Material Cycling, Nanjing University, Nanjing 210023, China; Jiangsu Provincial Key Laboratory of Geographic Information Science and Technology, International Institute for Earth System Science, Nanjing University, Nanjing 210023, China; Key Laboratory of Atmospheric Environment and Extreme Meteorology, Institute of Atmospheric Physics, Chinese Academy of Sciences, Beijing 100029, China; Shanghai Engineering Research Center of Solid Waste Treatment and Resource Recovery, School of Environmental Science & Engineering, Shanghai Jiao Tong University, Shanghai 200030, China; School of Atmospheric Sciences, Guangdong Province Data Center of Terrestrial and Marine Ecosystems Carbon Cycle, Sun Yat-sen University, Zhuhai 519082, China; School of Atmospheric Sciences, Guangdong Province Data Center of Terrestrial and Marine Ecosystems Carbon Cycle, Sun Yat-sen University, Zhuhai 519082, China; School of Atmospheric Sciences, Guangdong Province Data Center of Terrestrial and Marine Ecosystems Carbon Cycle, Sun Yat-sen University, Zhuhai 519082, China; Department of Geography, The University of Hong Kong, Hong Kong 999077, China; Department of Atmospheric and Oceanic Sciences, School of Physics, Peking University, Beijing 100871, China; Institute of Energy, Environment and Economy, Tsinghua University, Beijing 100084, China; State Key Laboratory of Tibetan Plateau Earth System, Resources and Environment (TPESRE), Institute of Tibetan Plateau Research, Chinese Academy of Sciences, Beijing 100101, China; State Key Laboratory of Tibetan Plateau Earth System, Resources and Environment (TPESRE), Institute of Tibetan Plateau Research, Chinese Academy of Sciences, Beijing 100101, China; School of Atmospheric Sciences, Guangdong Province Data Center of Terrestrial and Marine Ecosystems Carbon Cycle, Sun Yat-sen University, Zhuhai 519082, China; Tianjin Key Laboratory of Water Resources and Environment, Tianjin Normal University, Tianjin 300387, China; School of Atmospheric Sciences, Guangdong Province Data Center of Terrestrial and Marine Ecosystems Carbon Cycle, Sun Yat-sen University, Zhuhai 519082, China; State Key Laboratory of Atmospheric Boundary Layer Physics and Atmospheric Chemistry (LAPC), Institute of Atmospheric Physics, Chinese Academy of Sciences, Beijing 100029, China; Jiangsu Key Laboratory of Atmospheric Environment Monitoring and Pollution Control, Collaborative Innovation Center of Atmospheric Environment and Equipment Technology, School of Environmental Science and Engineering, Nanjing University of Information Science & Technology (NUIST), Nanjing 210044, China; Carbon-Water Research Station in Karst Regions of Northern Guangdong, School of Geography and Planning, Sun Yat-Sen University, Guangzhou 510006, China; State Key Laboratory of Atmospheric Boundary Layer Physics and Atmospheric Chemistry (LAPC), Institute of Atmospheric Physics, Chinese Academy of Sciences, Beijing 100029, China; Key Laboratory of Coastal Environment and Resources of Zhejiang Province, School of Engineering, Westlake University, Hangzhou 310024, China; Institute of Advanced Technology, Westlake Institute for Advanced Study, Hangzhou 310024, China; Institute of Blue and Green Development, Shandong University, Weihai 264209, China; College of Geography and Remote Sensing, Hohai University, Nanjing 211000, China; Institute of Carbon Neutrality, Sino-French Institute for Earth System Science, College of Urban and Environmental Sciences, Peking University, Beijing 100871, China; Institute of Carbon Neutrality, Sino-French Institute for Earth System Science, College of Urban and Environmental Sciences, Peking University, Beijing 100871, China

**Keywords:** greenhouse gas budget, China, carbon dioxide, methane, nitrous oxide

## Abstract

National greenhouse gas (GHG) budget, including CO_2_, CH_4_ and N_2_O has increasingly become a topic of concern in international climate governance. China is paying increasing attention to reducing GHG emissions and increasing land sinks to effectively mitigate climate change. Accurate estimates of GHG fluxes are crucial for monitoring progress toward mitigating GHG emissions in China. This study used comprehensive methods, including emission factor methods, process-based models, atmospheric inversions, and data-driven models, to estimate the long-term trends of GHG sources and sinks from all anthropogenic and natural sectors in China's mainland during 2000–2023, and produced an up-to-date China GHG Budget dataset (CNGHG). The total gross emissions of the three GHGs show a 3-fold increase from 5.0 (95% CI: 4.9–5.1) Gt CO_2_-eq yr^−1^ (in 2000) to 14.3 (95% CI: 13.8–14.8) Gt CO_2_-eq yr^−1^ (in 2023). CO_2_ emissions represented 81.8% of the GHG emissions in 2023, while 12.7% and 5.5% were for CH_4_ and N_2_O, respectively. As the largest CO_2_ source, the energy sector contributed 87.4% CO_2_ emissions. In contrast, the agriculture, forestry and other land use sector was the largest sector of CH_4_ and N_2_O, representing 50.1% and 66.3% emissions, respectively. Moreover, China's terrestrial ecosystems serve as a net CO_2_ sink (1.0 Gt CO_2_ yr^−1^, 95% CI: 0.2–1.9 Gt CO_2_ yr^−1^) during 2012 to 2021, equivalent to an average of 14.3% of fossil CO_2_ emissions. Our GHG emission estimates showed a general consistency with national GHG inventories, with gridded and sector-specific estimates of GHG fluxes over China, providing the basis for curtailing GHG emissions for each region and sector.

## Teaser

A comprehensive accounting of greenhouse gas budget of China, including CO_2_, CH_4_ and N_2_O, was reported for the period of 2000–2023. The budget not only harmonized detailed accounting for more than 40 flux terms, but also got verification from atmospheric inversions.

## INTRODUCTION

Climate change is one of the greatest challenges to human survival and socio-economic development since the last century. Achieving net zero greenhouse gas (GHG) emission is fundamental in containing global warming [1]. Since the United Nations Framework Convention on Climate Change (UNFCCC) was officially adopted in 1992, the international community has prioritized GHG emission mitigation [2]. In particular, the Paris Agreement, adopted in 2015 by the Conference of the Parties (COP), set a quantified goal to limit temperature rise to 2°C by the end of this century compared to pre-industrial global mean temperature and strive to control at 1.5°C (https://unfccc.int/documents/184656). Subsequent COP meetings have highlighted the importance of the 1.5°C warming target. According to a synthesis report, in

order to achieve the 1.5°C temperature rise target, the world must achieve net zero CO_2_ emissions by 2050 (i.e. anthropogenic CO_2_ absorption exceeds anthropogenic emissions) and net zero GHG emissions by 2070 [1]. Successive COPs have called for countries to come up with more ambitious national climate action plans for achieving the warming target.

An accurate estimate of GHG emissions and sinks is the basis for countries to develop action plans for achieving GHG neutrality [3]. Therefore, the UNFCCC, adopted as early as 1992, obliges each Party to submit its own inventory of GHG sources and sinks. It requires Annex I countries (most developed countries) to submit National Communications every 4 years to provide the international community with a comprehensive understanding of national actions and progress in addressing climate change. COP 16 in 2010 and COP 17 in 2011 mandated that non-Annex I Parties submit biennial updated reports starting in 2014, in accordance with their capacity. Furthermore, COP 21 in 2015 required all Parties to submit inventories of GHG emissions every 2 years beginning in 2024. At the same time, the Paris Agreement stipulates that each country submits a national climate action plan (i.e. Nationally Determined Contribution) every 5 years. These plans serve as the basis for formulating an action plan and correspondingly conducts a global stocktake 2 years in advance. This stocktake is a comprehensive and facilitative assessment of the overall global progress in implementing the agreement with regard to mitigation, adaptation, implementation means, and support. However, the current international understanding of the state of GHG emissions is not satisfactory. Six of the 43 Annex I countries did not submit their national inventories as required, only 2 non-Annex I countries (total 154 countries) submitted 6 ‘national communications’, and only 36 submitted more than 4 (https://unfccc.int/non-annex-I-NCs#fn1). For example, China, as a non-Annex I country, submitted 4 national communications in 2004, 2012, 2019 and 2023. The submitted national GHG emission inventories still suffer from low transparency and integrity, as well as high uncertainty in emission estimates [4], which greatly limits their ability to serve the formulation of national GHG emission reduction plans.

While the need for national GHG budgets is urgent, what is available for scientists and policy makers remains insufficient. While numerous global emission datasets provide some sectorial GHG emissions at national, provincial and even grid scales, these datasets commonly make strong assumptions on activities and emission factors (e.g. Tier 1 of the IPCC inventory guidelines), leading to large regional biases. For example, the widely used EDGAR (Emissions Database for Global Atmospheric Research) dataset estimates CH_4_ emissions from energy extraction with high uncertainty in several energy-producing countries: it overestimates Russia's emissions by more than two times and underestimates emissions from Iraq, Kuwait and Qatar by nearly 80% compared to the national communications [5]. In addition, certain datasets employ their own sectoral classifications, and most of the datasets do not cover comprehensive sectors, particularly the agriculture, forestry and other land use sector (AFOLU), limiting the formulation of sectoral emission reduction plans [6]. Finally, only a few datasets include all three GHGs, namely CO_2_, CH_4_ and N_2_O, thus failing to meet the latest needs to address climate change and achieve GHG neutrality goals [1]. It is urgent to construct a new generation of accurate, comprehensive source-sink flux datasets that cover more sectors and include the three main GHGs, so as to provide data support for countries to formulate effective pathways to achieve GHG neutrality [7]. Community-based GHG accounting taking full advantage of process-based modelling, progressing analytical and artificial intelligence models should help improve both the accuracy and transparency of national GHG budgets, given state-of-the-art reporting infrastructure.

China is both among the largest GHG emitters and a global leader in promoting nature-based solutions to mitigate climate change [6,8]. On the one hand, China's industrialization in the past few decades has significantly increased its energy consumption and CO_2_ emissions [9], and as a major agricultural country with extensive rice cultivation areas and substantial nitrogen fertilizer application [10,11], China generates significant CH_4_ and N_2_O emissions [11]. On the other hand, China has implemented the world's largest-scale ecological engineering since the last century which has significantly enhanced the carbon sink function of terrestrial ecosystems [8,12]. China is becoming a major stakeholder in international climate governance and a major advocate of climate change mitigation efforts. However, harmonized accounting for both anthropogenic emissions and terrestrial ecosystem emissions and sinks in the comprehensive GHG budget has been lacking. In this study, we used both the atmospheric inversions (known as the ‘top-down’ approach) and the ground-based inventories (known as the ‘bottom-up’ approach) to calculate emissions and sinks of the three greenhouse gases (CO_2_, CH_4_ and N_2_O) across all four sectors in China: energy, industrial processes and product use (IPPU), AFOLU and waste. The estimates, derived from a combination of emission factor methods, process-based ecosystem models and data-driven models, produced a dataset named the China Greenhouse Gas Budget (CNGHG), covering the period from 2000 to 2023.

## RESULTS

**Figure 1.**
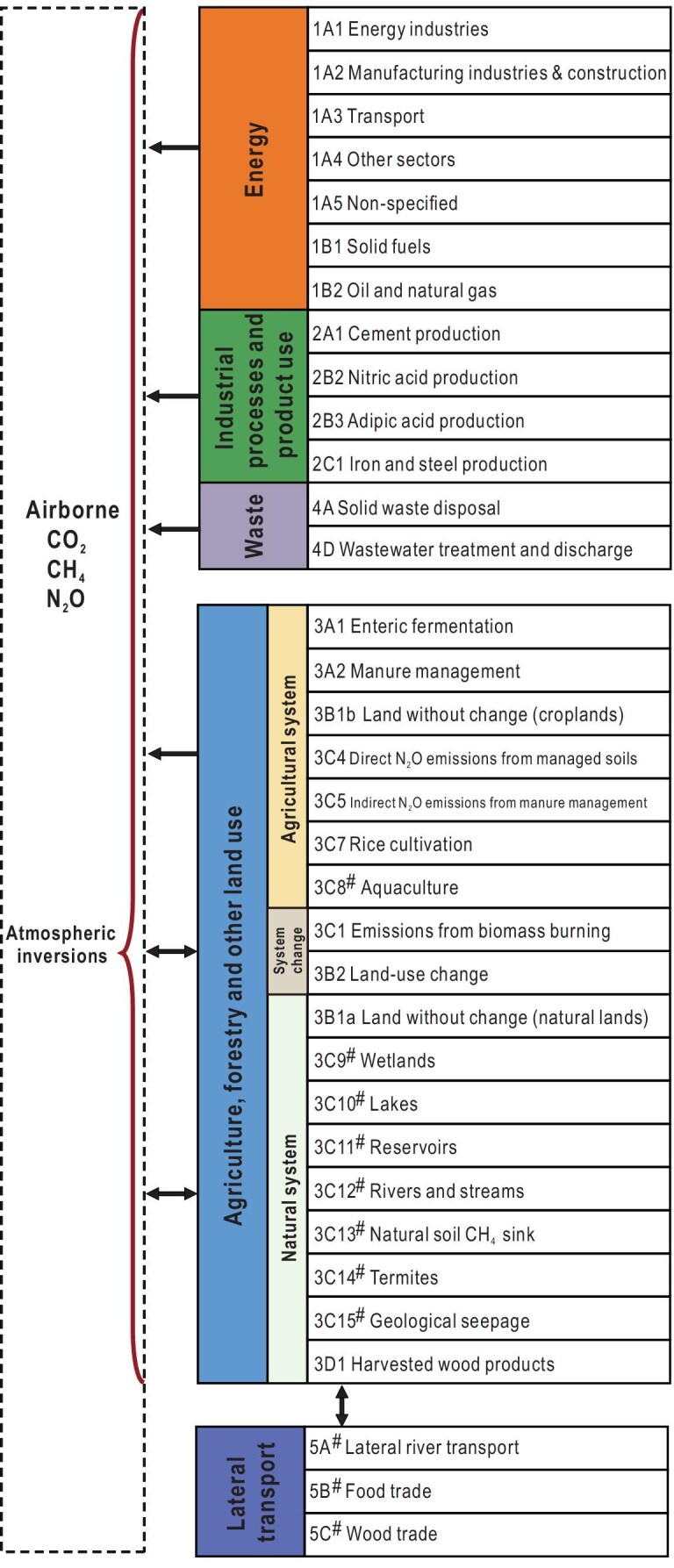
Accounting framework of the greenhouse gas budget with dual constraints. The code corresponds to the categories in the 2019 Refinement to the 2006 IPCC Guidelines on National Greenhouse Gas Inventories. ^#^These categories are newly added in this study and not included in the 2019 Refinement. More details about the definitions and methodologies of each sector can be found in Tables S1–S3.

The assessment of China's GHG budget aligns consistently with the methodology and terms used by the Global Carbon Project and REgional Carbon Cycle Assessment and Processes (RECCAP) proposal [13], which contains a set of shared and agreed-upon definitions that are as precise as possible for reporting each CO_2_ flux. This study further established the accounting framework to encompass three key greenhouse gases based on Wang *et al.* [14]. The definitions of flux terms for each sector are aligned with the IPCC guidelines for easier utilization by policy makers and have also included some terms of fluxes that have not yet been incorporated in IPCC guidelines (Fig. 1). The GHG budget is constrained by both observation-based assessments from atmospheric inversions of GHG mixing ratios (the top-down approach) and land-based assessments based on inventories, model simulations of carbon storage change, and model estimates of GHG fluxes (the bottom-up approach), which makes it a verifiable accounting. This dual constraint approach is designed to facilitate cross-validation of the findings from both the top-down and bottom-up methodologies, as well as other independent estimates (Fig. 1).

### Change of emissions and sinks since 2000

**Figure 2.**
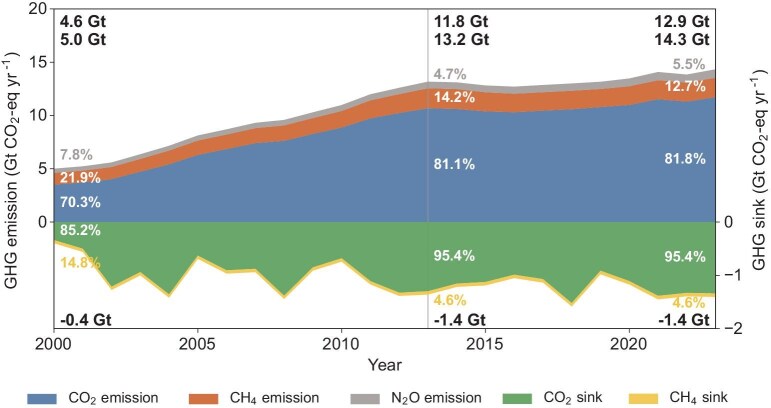
Temporal variations in greenhouse gas (GHG) emissions and sinks from 2000 to 2023. The numbers of three lines from top to bottom in black bold font represent the net emissions, gross emissions and land sinks in 2000, 2013 and 2023, respectively. The percentage shows the ratio of each greenhouse gas to total emissions or sinks. Different scales on the y-axes are used to clearly display the magnitude for GHG emissions and sinks, respectively.

From 2000 to 2023, the total GHG emissions and land sinks of China showed substantial variation. Gross emissions increased ∼2.86 times from 2000 (5.0 Gt CO_2_-eq yr^−1^, 95% CI: 4.9–5.1 Gt CO_2_-eq yr^−1^) to 2023 (14.3 Gt CO_2_-eq yr^−1^, 95% CI: 13.8–14.8 Gt CO_2_-eq yr^−1^) (Fig. 2). This study included CO_2_ and CH_4_ sinks of terrestrial ecosystems in China. Although land sinks also increased largely to 1.4 (95% CI: 0.4–2.4) Gt CO_2_-eq yr^−1^ in 2023 from 0.3 (95% CI: −0.6–1.2) Gt CO_2_-eq yr^−1^ in 2000, the net emissions still increased ∼2.8 times, reaching 12.9 (95% CI: 11.8–14.0) Gt CO_2_-eq yr^−1^ in 2023. We used a piecewise linear regression method to identify if there is a turning point in trends of gross emissions through the past 24 years. The results showed both gross and net GHG emissions experienced sharp increases before 2013, but increased slightly after 2013 (Fig. 2). Specifically, during the first 14 years (2000–2013), both gross and net GHG emissions increased with annual increase rates of 0.62 Gt CO_2_-eq yr^−1^ and 0.55 Gt CO_2_-eq yr^−1^, respectively. The second stage spanning from 2013 to 2023, witnessed low increase rates of 0.12 Gt CO_2_-eq yr^−1^ and 0.11 Gt CO_2_-eq yr^−1^, respectively. Among the three GHGs, the share of CO_2_ to total emissions increased from 70.3% in 2000 to 81.8% in 2023, while the shares of CH_4_ and N_2_O sources were 12.7% and 5.5%, respectively, in 2023 (Fig. 2). In addition, with the increase of land carbon sink, the share of CH_4_ sink showed a decreasing trend, representing 4.6% in 2023.

**Figure 3.**
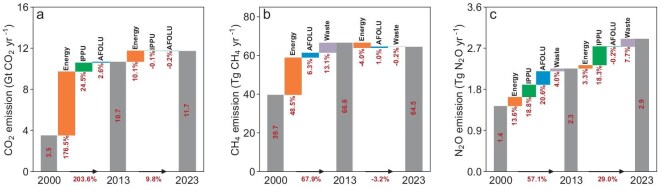
The contributions of each sector to the overall CO_2_ (a), CH_4_ (b) and N_2_O (c) emissions from 2000 to 2023. The three stacked columns represent the emissions in 3 years, with percentages depicted above the suspended columns indicating the contribution of each sector to the total percentage increase (black arrows). IPPU indicates industrial processes and product use, and AFOLU indicates agriculture, forestry, and other land use.

The sectoral profile of GHG emissions varied largely from 2000 to 2023 (Fig. S1). During the period of 2013–2023, CO_2_ emissions showed a lower increase rate (9.8%) compared to that of 2000–2013 (203.8%) (Fig. 3a). The energy sector played a pivotal role in the slowdown of CO_2_ growth, with its increase rates dropping to 10.1% during 2013–2023 compared to 176.6% for the period 2000–2013 (Fig. 3a). For CH_4_ emissions, there is a marked deceleration in the growth rate across nearly all sectors (Fig. 3b), and the largest rate of decrease was found in the energy sector from an increased rate of 48.5% in 2000–2013 to −4.0% during 2013–2023 (Fig. 3b). Similarly, the N_2_O emissions during 2013–2023 showed a lower increase rate (29.0%) than that of 2000–2013 (57.1%) (Fig. 3c). The AFOLU sector acted as the primary contributor, with its emission increasing by 20.6% of 2000–2013, but showing a low increase trend (−0.2%) (Fig. 3c). Notably, the increased rate of IPPU showed a similar upward trend during 2013–2023 (18.3%) with that of 2000–2013 (18.8%) (Fig. 3c).

This study also estimated lateral carbon fluxes resulting from the wood and food trades as well as the riverine-carbon export to the ocean and other countries (Table S1). The carbon flux included in the imported wood products was larger than that of exported wood products through the past two decades (Fig. S2a), and the differences also showed an increased trend at the rate of 0.0025 Gt CO_2_ yr^−2^ through the past two decades. Similarly, for the food trade, China showed a net imported carbon increase, and the net imported carbon flux increased more than 21 times from 0.029 Gt CO_2_ yr^−1^ in 2000 to 0.351 Gt CO_2_ yr^−1^ in 2021 (Fig. S2b). In addition, soil organic carbon was transported laterally to the ocean and other countries at the rate of 0.057 Gt CO_2_ yr^−1^ and 0.025 Gt CO_2_ yr^−1^ over the past two decades, respectively (Fig. S2c).

### Verification by the atmospheric inversions

As recommended by the revised IPCC guidelines for GHG inventories, our study employed atmospheric inversions to estimate the national GHG budget for three gases, which, along with bottom-up estimates, comprise the dual-constraint approach. Our atmospheric inversions show that China's land carbon sink was 1.14 ± 0.50 Gt CO_2_ yr^−1^ during 2015–2022, net CH_4_ emissions were 1.69 ± 0.13 Gt CO_2_-eq yr^−1^ during 2011–2022 and net N_2_O emissions were 0.54 Gt CO_2_-eq yr^−1^ during 2009–2022. By adjusting for lateral fluxes, the CNGHG narrows the gap between top-down and bottom-up estimates to within 20%, with top-down CO_2_ sink and CH_4_ balance aligning with bottom-up estimates within 10%, and the N_2_O difference around 17% (Fig. S3). This indicates that our estimates are verifiable within the uncertainties of either approach.

### Sectoral GHG source profiles in China

**Figure 4.**
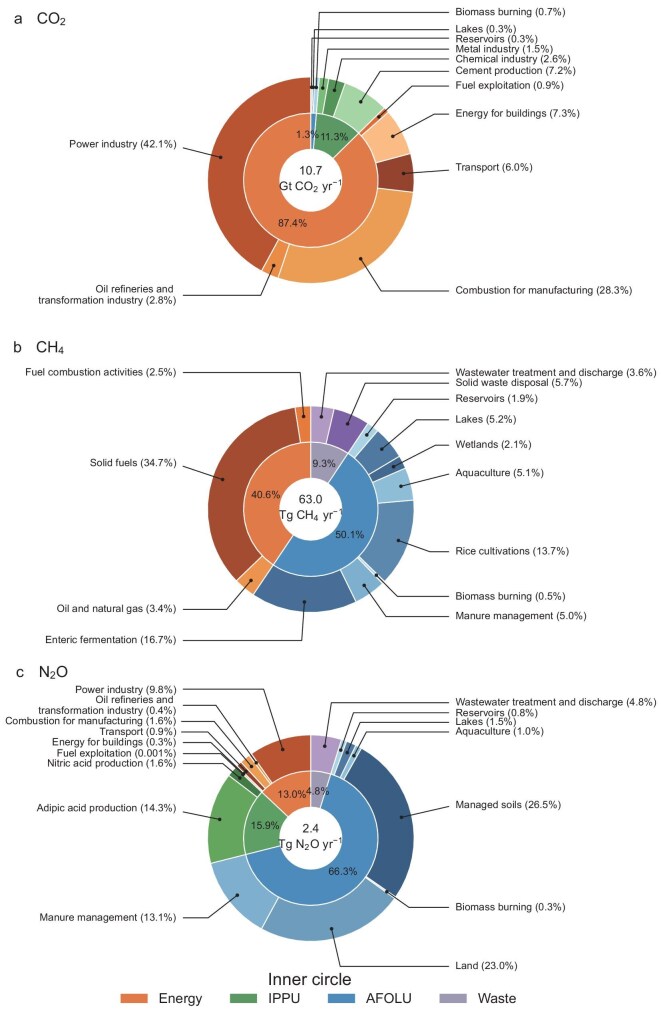
(a–c) Average sectoral shares of GHG sources in China over 2012–2021. IPPU indicates industrial processes and product use, and AFOLU indicates agriculture, forestry, and other land use. Emissions of the three GHGs across the four sectors are detailed in Table S4.

Based on the mean GHG emissions from 2012 to 2021 (10 years with statistical data available for all sectors), we analyzed the sectoral shares of GHG sources in China. The energy sector was the largest contributor to CO_2_ emissions, accounting for 87.4% of emissions (Fig. 4a). Notably, the power industry was the largest sub-sector within the energy sector (Fig. 4a). For CH_4_ emissions, the AFOLU sector was the largest sector of CH_4_ emissions, representing 50.1% emissions. The energy sector is the second largest contributor, and its share (i.e. 40.6%) was notably lower than its share for CO_2_ emissions (Fig. 4b). Within the AFOLU sector, enteric fermentation and paddy rice cultivation were two important sources, contributing 16.7% and 13.7% to the total CH_4_ emissions, respectively. Exploitation from solid fuels in the energy sector was the largest sub-sector (34.7%). For N_2_O emissions, the AFOLU sector accounted for 66.3% of N_2_O emissions, and the IPPU, energy and waste sectors contributed 15.9%, 13.0% and 4.8% of emissions, respectively (Fig. 4c). Managed soils in agriculture accounted for 26.5% of the total N_2_O sources, followed by land (23.0%) and adipic acid production (14.3%) (Fig. 4c).

### Spatial profile of GHG emissions and sinks in China

**Figure 5.**
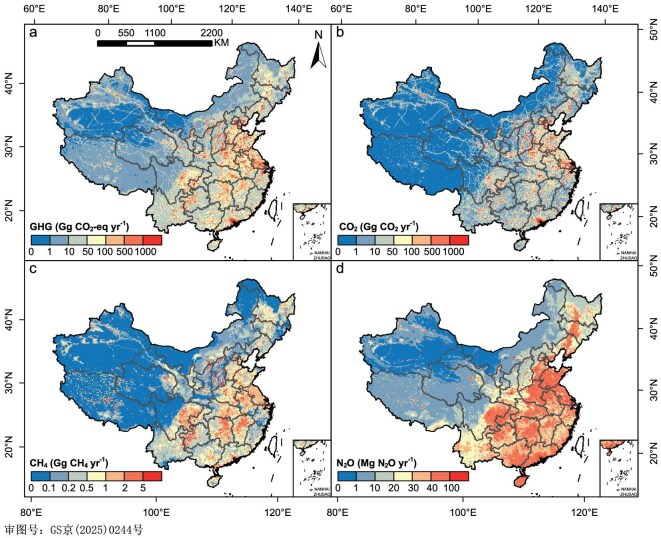
Spatial distributions on gross emissions of the total greenhouse gas (a), CO_2_ (b), CH_4_ (c) and N_2_O (d) in China averaged through 2012–2021. The values indicate the emissions of each pixel with an area of 100 km^2^. Data for Taiwan province is not included.

Gross GHG emissions differed largely across spatial regions, with significantly higher GHG emissions in economically developed regions (Fig. 5a). For example, Beijing-Tianjin-Hebei is one of the hotspots for GHG emissions, mostly because of large CO_2_ emissions (Fig. 5b). Significant point sources of CO_2_ and CH_4_ emissions include power plants and coal mines (Fig. 5 and Figs. S4–S5). Rice planting areas in Northeast and South China were the main contributors to CH_4_ emissions (Fig. 5c and Fig. S5). N_2_O emissions come prominently in areas with concentrated farmland distribution, such as North China, Southwest China and South China (Fig. 5d and Fig. S6).

To evaluate the terrestrial carbon sink, 6 process-based models were used in this study, which were driven by the same land cover change and climate datasets (Table S1 and Section S1.3). The mean net biome production (NBP) derived from 6 models was 1.20 ± 0.19 Gt CO_2_ yr^−1^ averaged from 2012 to 2021 (Fig. S7a). There was a large spatial heterogeneity of simulated NBP over the entire study area with high carbon sinks in Northeast, South and Southwest China, and low carbon sinks in Northwest China (Fig. S7a). The wildfires resulted in CO_2_ emissions of 0.076 ± 0.025 Gt CO_2_ yr^−1^ during the same period, and the soil organic carbon was transported laterally to the ocean and other countries at the rate of 0.058 ± 0.002Gt CO_2_ yr^−1^ and 0.025 ± 0.003 Gt CO_2_ yr^−1^. Therefore, the NBP in China was 1.041 ± 0.201 Gt CO_2_ yr^−1^. A semi-empirical model revealed a soil CH_4_ sink of 0.06 Gt CO_2_-eq yr^−1^ over the whole of China from 2012 to 2021 (Fig. S7b). In addition, the top-down atmospheric inversion method captures similar spatial emission hotspots for the three greenhouse gases, further supporting the verifiability of our estimates (Figs S8–S10).

### Ratio of land carbon sink for offsetting emissions

Based on our estimates, we can also quantify the ratio of CO_2_ emissions from the energy and IPPU sectors that was offset by land carbon sink. Averaged from 2012 to 2021, the total land carbon offset about 14.34% of fossil CO_2_ emissions in China. It should be noticed that there was a large heterogeneity of offset ratio among various provinces (Fig. S11). This study included 31 provinces, and only in Xizang the carbon sink of terrestrial ecosystem has surpassed the energy and IPPU CO_2_ emissions (i.e. carbon neutrality) (Fig. S11). In addition, Yunnan, Heilongjiang, Guangxi, Guizhou, Sichuan and Qinghai also showed a high offset ratio of terrestrial carbon sink to CO_2_ emissions (Fig. S11). In several provinces of North, East and South China, the offsetting ratios of terrestrial carbon sink were <10% (Fig. S11).

### Comparison with national GHG inventories

**Figure 6.**
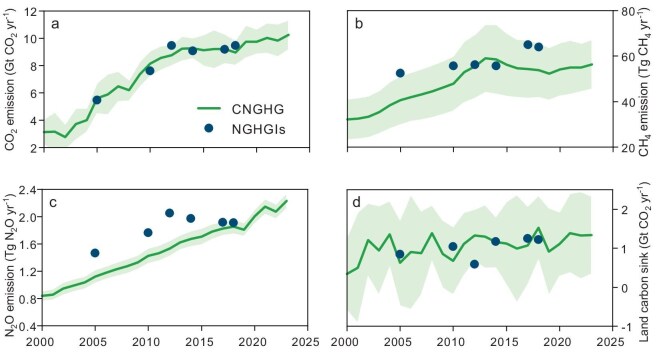
Comparison of estimated emissions and sinks by this study (i.e. CNGHG) with National Greenhouse Gas Inventories (NGHGIs). (a–c) indicate gross CO_2_, CH_4_ and N_2_O emissions, and (d) indicates land carbon sink. The gross greenhouse gas emissions and land carbon sinks presented in this figure are calculated using the same sub-sectoral categories as those in the NGHGIs. The shadow area indicates the uncertainty range within the 95% confidence interval of each sector.

Our estimates of GHG sources and sinks (i.e. CNGHG dataset) were generally comparable in magnitude with National GHG Inventories (NGHGIs) for the years 2005, 2010, 2012, 2014, 2017 and 2018 (Fig. 6). The mean CO_2_ emissions from the energy, IPPU and waste sectors derived from CNGHG for these 6 years were 8.33 ± 1.39 Gt CO_2_ yr^−1^, which were 1.11% lower than those of the NGHGIs (8.42 ± 1.56 Gt CO_2_ yr^−1^) (Fig. 6a). It should be noticed that there were consistent CH_4_ emissions between the CNGHG and NGHGIs in 2010, 2012 and 2014, but significant differences were observed in 2005, 2017 and 2018 (Fig. 6b). The comparison also indicated lower estimates of total N_2_O emissions over almost all 6 years by the CNGHG dataset compared with NGHGIs (Fig. 6c). The results showed high consistency in land carbon sink estimates (1.08 ± 0.31 Gt CO_2_ yr^−1^ for 6 years), which were averaged by 6 process-based models with NGHGI data (1.01 ± 0.26 Gt CO_2_ yr^−1^) (Fig. 6d).

## DISCUSSION

### Comparisons of CNGHG with NGHGIs and other datasets

This study estimated CO_2_, CH_4_ and N_2_O budgets across all sectors from 2000 to 2023 in China and produced a comprehensive dataset (i.e. CNGHG). Notably, the CNGHG dataset not only covers anthropogenic emissions, but also includes sources and sinks of terrestrial ecosystems, which are crucial for quantifying the global and regional GHG budget [6]. In comparison, existing GHG emission datasets concentrate on the energy and IPPU sectors, often overlooking the AFOLU sector [9]. Datasets like Carbon Monitor, CEADs (Carbon Emission Accounts and Datasets), ODIAC (Open-Data Inventory for Anthropogenic Carbon dioxide) and MEIC (Multi-resolution Emission Inventory for China) do not include sources and sinks for the AFOLU sector [15–17]. Although several datasets include the AFOLU sector, such as CEDS (Community Emissions Data System) and PRIMAP (Potsdam Realtime Integrated Model for probabilistic Assessment of emissions Paths), they only include enteric fermentation and manure management while ignoring other emission sources and all sinks [18,19]. The EDGAR dataset includes most sources of AFOLU but omits land sink and fluxes from land use changes [20]. In contrast, the CNGHG dataset includes detailed categories of energy, IPPU, AFOLU and waste sectors. Moreover, it introduces several new emission sources (e.g. inland water) that are seldom included in existing datasets (Tables S1–S3).

Besides encompassing more comprehensive sectors, the CNGHG dataset employs higher-tier methods and emission factors to quantify GHG emissions. The IPCC guidelines define estimation methods as 3 tiers [21]. However, most global emission datasets use Tier 1 methods, i.e. using global constant emission factors, which may result in large uncertainties [5]. In contrast, the CNGHG dataset primarily utilizes Tier 2 and Tier 3 methods, which largely improved the estimation accuracy (Tables S1–S3). For example, previous studies have highlighted that the emission factors for coal-related sources recommended by the IPCC are larger than the actual emission factors in China [15]. This study uses emission factors for energy and industry CO_2_ emissions derived from provincial guidelines for greenhouse gas inventories provided by the Chinese government (Tier 2) [22]. Moreover, several newly developed datasets are used to identify the emission locations (Section S1), further improving estimate accuracy in terms of spatial distribution. For example, the CNGHG dataset used a satellite-based high-resolution distribution dataset of paddy rice cultivation to estimate CH_4_ emissions [10], which performed well in identifying paddy rice locations. In contrast, existing datasets (i.e. EDGAR) used a time-invariant paddy rice distribution for the year 2000 to estimate CH_4_ emissions [23], which may lead to large uncertainties.

The GHG emissions were quite similar to those derived from the NGHGIs (Fig. 6), though many details of the NGHGIs are not available to the community. For example, energy CO_2_ emissions derived from the CNGHG dataset were quite close to the NGHGIs (Table S8) because the CNGHG dataset used provincial emission factors suggested by the Chinese government [22]. It should be noticed that our estimates for the AFOLU sector were also quite close to the NGHGIs (Table S8), despite different methods being used [24–26]. For example, the CNGHG dataset used 6 process-based ecosystem models to quantify land sinks, whereas the NGHGIs used IPCC guidelines. Nonetheless, the estimated terrestrial carbon sinks from CNGHG and NGHGIs were very close (Fig. 6d; Ref [26]). In contrast, the estimated global carbon budget largely underestimated the carbon sink of the terrestrial ecosystem in China [26]. One of the key reasons is that the CNGHG used a more accurate land use change dataset in China developed by Xia *et al.* [27], which substantially corrected the errors in the land use dataset used by the global carbon budget. The development of the CNGHG dataset is crucial for addressing critical data gaps and improving the accuracy of greenhouse gas estimates. Beyond its methodological advancement, this dataset offers a significant advantage by providing gridded, time-continuous data that can be updated annually, enabling more dynamic and reliable assessments.

### Spatial and temporal variations of GHG emissions

Our estimates revealed substantially temporal variations in GHG emissions during the past decades. A previous study showed a slow increasing trend of CO_2_ emissions during the 1980s and 1990s, followed by a rapid increase over the subsequent decade until 2010, and then exhibited a slower growth rate since 2010 [9]. Our estimates supported this conclusion that the CO_2_ emissions have reached a ‘peak plateau’ stage, a critical transition towards achieving carbon peaking [9,28]. As a dominant source, the energy sector was driven by a large consumption of coal in the 2000s (Fig. S12). Starting from the early of 2010s, a series of policies were implemented by the Chinese government to reduce coal consumption [29,30]. For example, driven by energy transition policies, the share of clean energy consumption reached 26.4% in 2023, marking an increase of 10.9% compared to 2013, while the share of coal consumption decreased by 12.1% (white paper on China's energy transition, http://english.scio.gov.cn/whitepapers/2024-08/29/content_117394384.htm). Consequently, the increase of CO_2_ emissions in the energy sector has slowed down during the 2010s. Similarly, the CH_4_ emissions of the energy sector saw stagnant increases in CH_4_ emissions due to reductions in coal usage since the 2010s [31].

Although the AFOLU sector constitutes a relatively small portion of the CO_2_ budget, our estimates reveal a significant increasing trend in terrestrial carbon sinks. Over the past decades, China has launched several ecological restoration initiatives, such as the Three North Shelter Forest Program, the Natural Forest Protection Program, and the Grain for Green project, all of which have contributed to nationwide forest area growth. The land use change dataset used to drive six ecosystem models, sourced from Xia *et al.* [27], shows that China's forested area has doubled over the past 40 years. A recent study suggested that ∼72.7% of the increase in land carbon sinks can be attributed to these ecological restoration programs [32], highlighting the significant role of active land management in achieving carbon neutrality in China.

N_2_O emissions of different sectors displayed varying trends. Specifically, N_2_O emissions of the AFOLU sector have decreased during recent years, while emissions from the other sectors increased (Fig. 3c). In the agricultural sector, N_2_O emissions initially increased, peaking in 2013, and then declined thereafter. The reduction observed between 2013 and 2023 is primarily attributed to a decrease in nitrogen application intensity (Fig. S13), which significantly contributed to the decrease in N_2_O emissions from the AFOLU sector. Liang *et al.* [11] also documented comprehensive changes in N_2_O emissions among various sectors and highlighted the importance of national policies on reducing fertilizer use starting from 2017 which contributed largely the decreases of the AFOLU sector.

Our dataset also highlighted the significant spatial heterogeneity of GHG emissions. Each province needs to develop individualized roadmaps to mitigate its GHG emissions and achieve the goal of carbon neutrality [33]. Investigating the spatial heterogeneity of emissions is a crucial first step in this process because it is quite important for developing effective region-specific GHG mitigating policies [34]. Our results indicated that the shares of the three GHGs to total emissions vary significantly among provinces (Fig. S14a). For example, the share of CH_4_ to total GHG emissions was ∼30% in Shanxi province, driven by large coal production which was considerably higher than the national average. In addition, there were considerable differences in sectoral contributions across provinces (Fig. S14b). These variations in emissions played a crucial role in determining the diverse mitigation measures adopted by different provinces. Furthermore, previous studies have also shown substantial spatial differences in the ratio between land carbon sink and anthropogenic emissions [9]. Our results supported this conclusion that there is significant heterogeneity in the sink ratios to anthropogenic emissions. Moreover, high spatial variability in the potential for increased carbon sinks was observed, highlighting the need for coordinated emissions and mitigation schemes tailored to each province [8].

### Implications for evaluating processes of carbon neutrality in China

This study provides long-term data on CO_2_ sources and sinks, which is useful for evaluating the progress of carbon neutrality. China set a goal to reduce the emission intensity (i.e. induced anthropogenic CO_2_ emissions by per gross domestic product) by 40%–45% in 2020 compared to 2005. Previous research showed that carbon emission intensity had decreased by over 45% in 2020 compared to 2005, highlighting that China had exceeded its commitment [9,35]. Our results corroborated this conclusion that emission intensity showed a 46.24% decrease in 2020 compared with 2005 levels (Fig. S15). In addition, to achieve carbon neutrality, the Chinese government has set a plan that the emission intensity in 2030 needs to decrease by 65% compared to 2005 levels [35]. To achieve the 2030 target, the emission intensity should decrease at a rate of 3.80% yr^−1^ from 2024 to 2030, which is lower than that of 2010–2019 but higher than that of 2020–2023 (Fig. S15), implying that large efforts are still needed to achieve the carbon peak target.

Besides CO_2_, CH_4_ and N_2_O emissions have also received increasing attention. Achieving the 1.5°C temperature rise target requires reducing annual GHG emissions by ∼43% by 2030 at the level of 2019, and achieving net-zero GHG emissions by 2070 [1]. Therefore, it is crucial to evaluate CH_4_ and N_2_O emissions. Recently, the Chinese government stated that the 2035 nationally determined contributions will cover CH_4_ and N_2_O besides CO_2_ aligning with the call in COP 28 (Readout on Meeting of the U.S.-China Working Group on Enhancing Climate Action in the 2020s). The CNGHG dataset provided comprehensive data on sources and land sinks of CH_4_ and N_2_O covering all sectors, which could be a reference source for developing mitigation policies in the near future.

### Reducing estimate uncertainties

Quantifying and reducing uncertainties is a critical step in improving the accuracy of greenhouse gas budget estimates. To address this, we employed multiple models to estimate land carbon sinks, CH_4_ emissions from wetlands and rice paddies, and natural soil N_2_O emissions. Differences in model structures and parameters led to variations in the results. For example, estimates of land carbon sinks from six ecosystem models for 2012–2021 ranged from 0.73 to 1.75 Gt CO_2_ yr^−1^, highlighting the high uncertainties of relying on a single model. Averaging the model outputs, we estimated China's land carbon sink at 1.04 Gt CO_2_ yr^−1^, which aligns closely with results from atmospheric inversion approaches and NGHGIs. Similarly, CH_4_ emissions from rice paddies ranged from 6.48 to 10.85 Tg CH_4_ yr^−1^, wetland CH_4_ emissions from 1.09 to 1.52 Tg CH_4_ yr^−1^, and natural soil N_2_O emissions from 0.43 to 0.69 Tg N_2_O yr^−1^. These ranges reflect the complexity of biogeochemical processes and the influence of environmental parameters, further emphasizing the importance of integrating multiple models to reduce biases.

Our estimates provided insights into the GHG budget in China, but there are still uncertainties that should be noticed. On the ‘bottom-up’ side, the uncertainties primarily stem from activity data and emission factors related to anthropogenic GHG sources. The activity data of most sectors can be acquired from the various statistical yearbooks with low uncertainties. However, national or provincial statistical yearbooks did not cover all emission sources and the activity data of several sectors can only be acquired from literature or relevant databases, which did not include complete records (Table S9). For example, the national statistical system did not include the production of adipic acid and nitric acid, despite their significant contributions to the overall N_2_O emissions [11]. Therefore, this study highlighted that the national statistical system should include more GHG emission sources to improve the estimate accuracy of GHG budgets (Table S9). On the ‘top-down’ side, both prior information and the observational constraints could be improved in future studies, as well as how to minimize potential spatial representative issues in utilizing the observations for atmospheric inversions [36].

In addition, CNGHG provided gridded emissions of GHG for all sectors. Although our dataset integrated several new datasets on the location of multiple sources for estimating gridded emissions, there are remaining uncertainties regarding the emission locations for several sources. First, these location datasets did not include all emission sources of each sector. Second, these datasets did not provide comprehensive production information for each source. For example, this study used a comprehensive dataset of cement plants to allocate CO_2_ emission from cement production into the grids [37]. However, this dataset only provided the capacity of cement production for nearly half of those plants. Therefore, future studies are urgently needed to further develop new location datasets to improve the estimates of emission sources.

Overall, our CNGHG effort provides a pioneering example of the accuracy and transparency of national GHG budgets that could be achieved under the current infrastructure of data and models of the biogeochemical research community. This experience could be extended to other countries where monitoring systems are growing but lacking systematic GHG accounting, which is necessary to design both national and global pathways towards effective climate mitigation and climate neutrality.

## METHODS

Our estimates included the sources and sinks of three GHGs (i.e. CO_2_, CH_4_ and N_2_O) for four sectors: energy, industrial processes and product use (IPPU), agriculture, forestry and other land use (AFOLU) and waste. Specifically, the estimated sources and sinks covered multiple sub-sectors of the above four sectors, and followed the IPCC guidelines for national GHG inventories [21] (Tables S1–S3). To ensure accuracy, the estimates covered the period of 2000–2023 when statistical data was available for most emission sources. Comprehensive methods were used in this study, including emission factor methods, process-based ecosystem models, atmospheric inversion models and data-driven models (Tables S1–S3).

### Emission factor methods

Emission factor methods combine activity data (*AD*) and emission factor (*EF*) to estimate total emissions (*E*). *AD* means the consumption of a given fossil fuel or the extent of industry activity, and *EF* indicates the emissions per unit of activity. The equation is:


(1) \begin{eqnarray*}
E = AD \times EF.
\end{eqnarray*}

Detailed calculations are introduced in the Supplementary online data (S1.1–1.4). Sources of activity data are described in Section S2.

### Process-based ecosystem models, data-driven models and atmospheric inversions

Several process-based ecosystem models were used to estimate the carbon sink of the terrestrial ecosystem in this study, CH_4_ emissions from paddy rice and wetland, natural N_2_O emissions and land CH_4_ sink (Tables S1–S3; Section S1.3). All of these models have been validated previously at site, regional and global scales. Land carbon sink is estimated by the mean simulations of six process-based ecosystem models (Section S1.3; Table S1) [26]. Lateral carbon transport caused by soil erosion and leaching in China along the land-river-ocean continuum was simulated by the land surface model ORCHIDEE-Clateral [38]. Three process-based models, including CH4MOD [39], IBIS-CH_4_ [40] and TRIPLEX-GHG [41], were applied to quantify CH_4_ emissions across paddy rice (only CH4MOD and IBIS-CH_4_) and wetland (Table S2; Section S1.3). The IBIS-MicN model was applied to quantify N_2_O emissions of forest and grassland ecosystems [42] (Table S3; Section S1.3).

Data-driven models were developed to estimate GHG emissions of lakes, reservoirs, natural soils, managed soils and nitrogen deposition in cropland (Tables S1–S3; Section S1.3). In addition, this study used three atmospheric inversion systems to estimate the land carbon sink, two inversion systems to estimate CH_4_ sectoral emissions and one inversion system for N_2_O emissions (Section S1.5).

### Uncertainty analysis

To ensure the robustness and credibility of the CNGHG estimates, we conducted a comprehensive uncertainty assessment for each component using appropriate methods. For estimates based on emission factor methods, uncertainties were derived from both activity data and emission factors following the IPCC guidelines. For sectors using data-driven models, we evaluated the uncertainty by optimizing model parameters through Monte Carlo simulations (with 5 000 runs) to obtain the 95% confidence interval of the uncertainty range. For fluxes based on multiple process-based ecosystem models, the standard error was calculated across models and converted to a 95% confidence interval [42]. Considering the independence of each sector, the total budget uncertainty was calculated by aggregating the uncertainties from each sector and greenhouse gas [43].

## Supplementary Material

nwaf069_Supplemental_File

## Data Availability

This study generated the CNGHG dataset which is available at https://carbon.pku.edu.cn/data/English/index.htm.
