# Supplementary material for: China's greenhouse gas budget during 2000–2023
Source: Natl Sci Rev. 2025 Feb 22;12(4):nwaf069. doi: 10.1093/nsr/nwaf069 (PMC11951099; doi:10.1093/nsr/nwaf069)
Supplement: nwaf069_Supplemental_File [file nwaf069_supplemental_file.docx]

Supplementary Information for China’s greenhouse gas budget during 2000-2023

Supplementary Data

S1. Introduction of methods

S1.1 Greenhouse gas (GHG) emissions of energy sector

CO_2_ emissions of energy sector were calculated in accordance the 2019 refinement to the 2006 IPCC guidelines for national GHG inventories ^[1]^ and the guidelines for the preparation of provincial-level GHG inventories of the National Development and Reform Commission (NDRC) of China methodology ^[2]^, and see following Eq.1.

$E_{i,j}={AD}_{i,j}\times{NCV}_{i}\times{CC}_{i}\times O_{i,j}$ (1)

where *E_i,j_* was the CO_2_ emissions of the *i*th fossil fuel in the *j*th sector. *AD_i,j_* was the magnitude of consumed fossil fuel derived from China Energy Statistical Yearbook. *NCV_i_* was the calorific value per unit of fossil fuel. *CC_i_* was the carbon content per unit of calorific value. *O_i,j_* was the oxidation rate of the fuel in the combustion process. This study used the default values of *NCV_i_*, *CC_i_* and *O_i,j_* provided by NDRC.

Due to outdated energy balance statistics (one-year lag), this study could estimate the CO_2_ emissions for all 44 energy sub-sectors from 2000 to 2022. There were total energy consumptions for entire country for 2023 and shares of coal, oil, natural gas and other energy types including nuclear, wind power, hydro power and solar power for 2023. Therefore, we estimated total CO_2_ emissions of the energy sector (not for each sub-sector) in 2023 based on a multiple linear regression:

$TE=a1\times Coal+a2\times Oil+a3\times Gas+a4\times Oe+a5$ (2)

where *TE* was total CO_2_ emissions for the entire energy sector. The *Coal*, *Oil*, *Gas* and *Oe* indicated the consumptions of coal, oil, natural gas and other energy, respectively. The *a1* to *a5* were regression coefficients. The statistical consumptions of the above four energy types and the corresponding estimated CO_2_ emissions based on energy balance statistics from 2000 to 2022 were used to calibrate the coefficients. Total emissions for 2023 were then estimated according to the regression equation developed as the above. To avoid overfitting, we employed a validation method where 80% of the years were randomly selected for calibrating the regression model, and the remaining 20% years were used for validation. This process was repeated 500 times. The mean estimates from these 500 iterations were used as the estimated emissions for 2023 for the entire energy sector. The validations performed during these 500 iterations showed the strong performance of this regression equation in estimating total CO_2_ emissions (Fig. S16).

This study used a gridded distribution derived from Emissions Database for Global Atmospheric Research (EDGAR) dataset to allocate national estimates into the grids by the following equation:

${Grid}_{i,j}=E_{j}\times\frac{{Grid\_EDGAR}_{i,j}}{{E\_EDGAR}_{j}}$ (3)

where *Grid_i,j_* was CO_2_ emission of the *j*th sub-sector at the *i*th grid each year. *E_j_* was national emission of the *j*th sub-sector each year. *Grid_EDGAR_i,j_* and *E_EDGAR_j_* indicated the gridded emission of the *j*th sub-sector at the *i*th grid and the total emission of the *j*th sub-sector from the EDGAR dataset.

For energy CH_4_ emissions, this study estimated fugitive emissions from fuels, including from the coal and the oil and natural gas system (Table S2). In the coal sector, we utilized the dynamic mine-level dataset built by ref ^[3]^ and expanded it to year 2022 following its “integrating-calibrating” methodology. This dataset developed by ref ^[3]^ contains mine-specific operational status, geolocation, emission factor (EF), and annual productions. For methane emitted during mining and post-mining processes, the total emissions are estimated by combining EFs and annual productions. For methane escaped from abandoned coal mines, the decay curves methodology raised by ref ^[4]^ and validated in China by ref ^[3]^ is adopted. In the oil and gas system, facility-level information is mainly collected from ref ^[5]^ (natural gas fields), ref ^[6]^ (oil fields), and Global Energy Monitor (https://globalenergymonitor.org) (long-distance pipelines). Methane emissions from these facilities are estimated by multiplying EFs and activity levels, where EFs are from ref ^[5]^ and China’s official greenhouse gas inventories ^[7, 8]^. Activity levels are yearly calibrated to ensure the provincial and national consistency with statistics. For emission sources without identifiable facility information, such as municipal gas pipelines and oil refinery plants, emissions are estimated at the national level and then down-scaled to the grid level using the distribution pattern of secondary industries GDP ^[9]^ as a proxy.

N_2_O emissions of energy sector were derived from Full-scale Annual N_2_O dataset (FAN2020) produced by ref ^[10]^. Briefly, FAN2020 dataset used emission factor methods followed the IPCC 2019 methodology ^[1]^, and included 11 sources of energy N_2_O emissions. The FAN2020 dataset provided province-level estimates, and this study used a gridded distribution derived from the EDGAR dataset to allocate provincial estimates into the grids (Eq. 3).

S1.2 GHG emissions of industrial processes and product use (IPPU) sector

Emissions induced by cement production originate from the process of producing cement clinker. Carbon emissions resulting from cement production are estimated from the clinker production and its emission factor. As clinker production are not as widely available as cement production data, this study used cement production to estimate clinker production. We collected data on cement production in China and cement clinker production for the available years from the official dataset of the China Cement Yearbook prepared by the China Cement Association and the National Bureau of Statistics, respectively. Using the available statistical data, the mean ratio of clinker to cement production was calculated and used to estimate the clinker production for the years without statistical clinker production. A global gridded distribution of cement plants developed by ref ^[11]^ was used to allocate national emissions into the grid, which provided geographic location of 1159 cement plants and the production of 588 plants, accounting for about 58.18% of cement production in 2023. We assumed a time-invariant production ratio of 588 plants with national production, and allocated 58.18% of emissions to these 588 plants according to their ratios with total national production each year. The remaining emissions were evenly allocated to other plants. In addition, the CO_2_ emissions of chemical and metal industries were estimated based on emission factor method (Eq. 1). The default emission factors by IPCC inventory guideline ^[1]^ were used for the chemical industry, and the suggested emission factors by the NDRC ^[2]^ were used for the metal industry. The productions of both chemistry and metal products were from China Industry Statistical Yearbook.

N_2_O emission of the IPPU sector were derived from FAN2020 dataset ^[10]^, including emissions resulting from the production of nitric acid and adipic acid from 1980 to 2021. The FAN2020 dataset used Tier 2 emission factor to estimate N_2_O emission from adipic acid production, and Tier 1 factor for nitric acid production. There are 15 adipic acid factories in China. This study collected the location and production information for each factory from commercial reports ^[12]^. Therefore, national emissions of adipic acid production were allocated into the grids. Nitric acid emission was allocated to the grids based on the gridded EDGAR emissions. In addition, to extend the estimates to 2023, we used statistical production data of nitric acid and adipic acid in 2022, and collected adipic acid production of 2023 from ref ^[12]^. Predicted production data of nitric acid by ^[13]^ were also used.

S1.3 GHG emissions from agriculture, forestry and other land use (AFOLU) sector

In this sector, we followed the IPCC guidelines ^[1]^ to separate into four sub-sectors: livestock (3A), land (3B), aggregate sources and non-CO_2_ emissions sources on land (3C), and other (3D) (Table S1-S3). This study added several new sources emissions (Table S1-S3).

Six process-based ecosystem models were used to estimate land CO_2_ sink in the 3B sub-sector, including BEPS (Boreal Ecosystem Productivity Simulator) ^[14]^, IBIS (Integrated Biosphere Simulator) ^[15]^, LPJ-GUESS (Lund-Potsdam-Jena General Ecosystem Simulator) ^[16]^, ORCHIDEE (Organising Carbon and Hydrology In Dynamic Ecosystems) ^[17]^, TRIPLEX-GHG ^[18]^ and interactive Model of Air Pollution and Land Ecosystems (iMAPLE) ^[19]^. In addition, a newly developed bookkeeping model (i.e., land-use change emissions, LUCE) was used to quantify carbon flux resulting from land cover change ^[20]^. All models used the same forcing datasets of climate (section S2.2) and land cover change. The land cover change dataset was developed by Xia et al. ^[21]^, who combined the National Forest Inventory with 20 land use and land cover datasets to create a new method for reconstructing historical land cover in China. This updated dataset accurately reflects the historical changes in forest cover, providing a reliable basis for estimating terrestrial carbon sinks. For a detailed introduction to the six process-based ecosystem models and bookkeeping model, as well as an analysis of their validity, please refer to Xia et al. (under review in this issue).

CH_4_ emissions from natural wetlands were simulated using three process-based models: CH4MOD_wetland_ ^[22]^, IBIS-CH_4_ ^[23]^ and TRIPLEX-GHG ^[18]^. CH4MOD_wetland_ is a biogeophysical model to simulate CH_4_ production, oxidation, and emission processes from natural wetlands, which has been completely calibrated and validated across Chinese natural wetlands ^[22]^. IBIS-CH4 is a fully coupled process-based model including three major processes of production, oxidation, and transport, and especially simulates microbial dynamics ^[23]^. Comparison with observations showed that the IBIS-CH4 model effectively captured both the magnitude and variability of CH_4_ emissions across most sites under varying environmental conditions ^[23]^. TRIPLEX-GHG is a dynamic global vegetation model and a process-based wetland CH_4_ emission module was integrated with considering the processes of production, consumption, three ways of transport for wetland CH_4_, as well as the process of dynamic water table and a specific plant function type for wetland ^[18]^. TRIPLEX-GHG model effectively simulates the magnitude and temporal patterns of CH_4_ emissions from natural wetlands, making it suitable for application to different wetland conditions ^[18]^. All of three models used the water table depth as a driving forcing, which was simulated by TOPMODEL ^[24]^.

The assessment of agricultural sources of GHG emissions include N_2_O from cropland, CH_4_ from rice paddy, and N_2_O and CH_4_ from livestock. N_2_O emissions from cropland were estimated by multiplying emission factor with nitrogen loads, which encompassed various items such as N fertilization, N deposition, N mineralization, N in crop residues, N fertilization in pasture, and N leaching. The data for nitrogen fertilization during 2000-2023 were derived from multi datasets and modified with National Bureau of Statistics of China ^[25]^. The EF for the first five items was derived from crop-specific data-driven models based on 1705 field observations, while the EF for N leaching was adopted from ref ^[26]^. CH_4_ emissions from rice paddies were simulated using two process-based models, IBIS-CH4 ^[23]^ and CH4MOD ^[27]^, driven by the same climate (section 2.2) and crop phenology data with consistent crop distribution. The IBIS-CH4 model simulates CH_4_ emissions from rice paddies by considering two major microbial processes: acetoclastic methanogenesis and hydrogenotrophic methanogenesis, and has demonstrated good performance in China. CH4MOD has shown its effectiveness in simulating seasonal CH4 emissions in rice paddies. For example, when validated with 94 field observations, the observed and simulated r² was 0.84, with a slope of 0.92 and an intercept of 41.1 ^[27]^. Furthermore, we provided a comprehensive assessment of China's livestock non-CO_2_ emissions based on the multi-source data foundation (official data and market-based crowdsourced data) of livestock population. This assessment covered 12 categories of animals and three sectors of non-CO_2_ emissions. The Tier 2 approach following the IPCC 2019 refinement of estimation was employed to incorporate inter-annual variations in livestock population and productivity. A gridded livestock distribution dataset (Gridded Livestock of the World) was used to allocate the provincial and county emissions to the grids ^[28, 29]^. The detailed estimation methods of agricultural non-CO_2_ GHG emissions were introduced by ref ^[25]^.

GHG emissions from lakes and reservoirs were estimated by utilizing a data-driven approach. To construct reliable models to estimate the areal GHG emission rate, we compiled 666,836 water quality records from 217 lake and reservoir monitoring stations across China from the China National Environmental Monitoring Center (CNEMC) (https://www.cnemc.cn/sssj/). For each gas (CO_2_, CH_4_ and N_2_O), we developed a combination of data-driven models and selected the most appropriate one that can best predict the areal emission rate from water quality parameters. Furthermore, the water surface area of both lakes and reservoirs varied substantially during the period 2000-2023 and exhibited huge spatial heterogeneity ^[30, 31]^. Therefore, based on the simultaneous water surface area of lakes and reservoirs over the study period, the annual fluxes of the three GHG emissions from China’s lakes and reservoirs were calculated separately, and the total fluxes, expressed in CO_2_ equivalent (CO_2_-eq), were computed by considering their respective greenhouse warming potential over 100-year time horizon. More details on the national assessment of GHG emissions from lakes and reservoirs are available in ref ^[32]^ in this issue.

S1.4 GHG emissions of waste sector

A bottom-up method was adopted to calculate the emissions of CH_4_ and N_2_O from waste sectors, including wastewater treatment plants (WWTP) and landfills. For landfills, the first-order decay model was employed, while Tier 3 methods were used for WWTP ^[1]^. These calculations were based on a database of the three-dimensional emission factors matrix and point source with detailed methods were introduced by ref ^[33]^ .

S1.5 Atmospheric inversions

This study used three atmospheric inversion systems to estimate land carbon sink, two inversion systems to estimate CH_4_ sectoral emissions (Table S5-6), and the inversion system PKU to assist the estimation of N_2_O balance. The national annual budget for the land carbon sink and CH_4_ emissions were estimated as the multi-model mean for corresponding tracers. A correction of 0.14 Pg C yr^-1^ was subtracted from the inversed land carbon sink, following the methodology described by ref ^[34]^ and ref ^[35]^. This correction addresses lateral carbon fluxes such as trade of crop and wood products, riverine-carbon export to the ocean, and biogenic non-CO_2_ volatile organic compounds. The detailed information of method and results were introduced by ref ^[36]^. For the N_2_O budget, we conducted Bayesian inversion using 7 surface in-situ observations to quantify N_2_O emissions in East Asia (95°E-133°E, 16°N-54°N) between 2009-2022. The GEOS-Chem 12.7.0 chemical transport model (<https://doi.org/10.5281/zenodo.3634864>) was used as the forward model to simulate the sensitivity of atmospheric N_2_O concentrations to emissions, which was driven by Modern-Era Retrospective Analysis for Research and Applications version 2 (MERRA-2) reanalysis meteorological fields with 0.5°×0.625° horizontal resolution. The boundary conditions were adjusted to match the latitudinal mean from global surface sites. The prior inventories included GFED4 ^[37]^ for biomass burning, ref ^[38]^ for ocean, and EDGARv6 for anthropogenic sources. To reduce the computational cost, we aggregated grid cells based on their sectorial emission characteristics and spatial location information, using the k-means algorithm, an approach often used by previous studies ^[39-41]^. Finally, our state vector included 146 elements. The optimized N_2_O emissions were obtained by minimizing a Bayesian cost function that balanced the information from observations and the prior emissions. Each grid cell included emissions contributions from all sectors, and we attributed posterior emissions to individual sectors based on contributions from these sectors in the prior inventory.

S1.6 Lateral transport

This study also included lateral carbon transport by international trade of food and woods as well as lateral river transport. The former two transports were estimated based on import and export statistical data of food and wood. This study collected import and export data of the wood products and food from FAO statistical database. The wood products included fuelwood and charcoal, industrial roundwood, sawnwood, wood-based panels, pulp, and paper and paperboard. The conversion coefficients recommended by IPCC (2019) ^[1]^ were used to calculate lateral carbon fluxes from forestry trade. Carbon content of each food type was used to calculate lateral carbon fluxes from food (Table S10). Lateral carbon transport from soils to the ocean and other countries through rivers was estimated using ORCHIDEE-Clateral ^[42, 43]^. The ORCHIDEE-Clateral model simulates lateral carbon fluxes, including both particulate and dissolved forms, through soil erosion and leaching processes, transporting carbon from terrestrial ecosystems to the ocean and other regions via inland water networks. It has been validated against observations in major European rivers, accurately simulating long-term average concentrations of total organic carbon and dissolved organic carbon in river flows ^[42]^. The lateral carbon transport flux estimated in this study are consistent with previous research ^[44, 45]^. The detailed information was reported by reference ^[46]^.

S2. Data sources

S2.1 Statistical data

Multiple statistical yearbooks were used in this study to acquire active data. Table S7 showed detailed information of used statistical yearbooks and the corresponding variables.

S2.2 Meteorological forcing data

To drive ecosystem models and data-driven models, we need a long-term series of meteorological forcing dataset covering from 1901 to 2023. This study developed a long-term series of meteorological forcing dataset (1901-2023, 0.1º×0.1º, 6-hour) by integrating two datasets of CRU-JRA 2.4.5 (1901-2022, 0.5º×0.5º, 6-hour) and ERA5-Land reanalysis (1950-2023, 0.1º×0.1º, 6-hour). Specifically, we first resampled CRU dataset to 0.1º×0.1º spatial resolution (CRU0.1). The correlation of each variable between CRU0.1 and ERA5 was calculated at each pixel at 6-hour temporal scale, and used the regression relationships and CRU0.1 dataset at each pixel to recalculate new values of all variables through 1901-1949. Totally, 13 meteorological variables are provided, including downward shortwave radiation, downward longwave radiation, surface pressure, relative humidity, total precipitation, snowfall, zonal component of wind speed, meridional component of wind speed, wind speed, air temperature, maximum air temperature, minimum air temperature, and dewpoint temperature.

**Table S1.** Sectors and categories of CO_2_ sources and sinks included in China Greenhouse Gas emission dataset (CNGHG).

| **Sector** | **Source** | **Method^1^** | **Code^2^** |
| --- | --- | --- | --- |
| Energy | Fuel combustion activities* |  | 1A |
|  | Energy industries* |  | 1A1 |
|  | Main activity electricity and heat production* |  | 1A1a |
|  | Electricity generation | EF (T2) | 1A1ai |
|  | Heat plants |  | 1A2aiii |
|  | Petropleum refining |  | 1A1b |
|  | Manufacture of solid fuels and other energy industries* |  | 1A1c |
|  | Manufacture of solid fuels | EF (T2) | 1A1ci |
|  | Gas production |  | 1A1cii |
|  | Manufacturing industries & construction | EF (T2) | 1A2 |
|  | Transport | EF (T2) | 1A3 |
|  | Other sectors* |  | 1A4 |
|  | Residential | EF (T2) | 1A4b |
|  | Agriculture/forestry/fishing/fish Farms |  | 1A4c |
|  | Non-Specified | EF (T2) | 1A5 |
| Industrial processes and product use | Mineral industry* |  | 2A |
|  | Cement production | EF (T2) | 2A1 |
|  | Chemical industry | EF (T1) | 2B |
|  | Metal industry* |  | 2C |
|  | Iron and steel production | EF (T2) | 2C1 |
| Agriculture, forestry and other land use | Land |  | 3B |
|  | Land without change | PM, BK, AI | 3B1 |
|  | Land-use change | PM, BK | 3B2 |
|  | Aggregate sources and non-CO_2_ emissions sources on land* |  | 3C |
|  | Emissions from biomass burning* |  | 3C1 |
|  | Emissions from biomass burning in forest lands | EF | 3C1a |
|  | Emissions from biomass burning in croplands |  | 3C1b |
|  | Emissions from biomass burning in grasslands |  | 3C1c |
|  | Emissions from biomass burning in other lands |  | 3C1d |
|  | Aquaculture# | EF | 3C8 |
|  | Lakes# | DD | 3C10 |
|  | Reservoirs# | DD | 3C11 |
|  | Rivers and streams# | DD | 3C12 |
|  | Harvested wood products | EF (T2) | 3D1 |
| Lateral transport | Lateral river transport# | EF | 5A |
|  | Food trade# | EF | 5B |
|  | Wood trade# | EF | 5C |

^1^Method abbreviation: EF (emission factor method), T1/2 (Tier 1/2), PM (process-based model), BK (book-keeping model), AI (atmospheric inversion) and DD (data-driven method). ^2^Code indicates the corresponding code of categories in the 2019 Refinement to the 2006 IPCC Guidelines on National Greenhouse Gas Inventories, and flux terms added in this study. *The emissions of these categories were not estimated directly, but the sum of their sub-categories. #These categories are newly added in this study not be included by in the 2019 Refinement to the 2006 IPCC Guidelines on National Greenhouse Gas Inventories.

Table S2. Sectors and categories of CH_4_ sources and sinks included in China Greenhouse Gas emission dataset (CNGHG).

| **Sector** | **Source** | **Method^1^** | **Code^2^** |
| --- | --- | --- | --- |
| Energy | Fuel combustion activities | EF (T2) | 1A |
|  | Fugitive emissions from fuels* |  | 1B |
|  | Solid fuels | EF (T2) | 1B1 |
|  | Oil and natural gas |  | 1B2 |
| Agriculture, forestry and other land use | Livestock* |  | 3A |
|  | Enteric fermentation* |  | 3A1 |
|  | Dairy Cows | EF (T2) | 3A1ai |
|  | Other Cattle | EF (T2) | 3A1aii |
|  | Buffalo | EF (T2) | 3A1b |
|  | Sheep | EF (T2) | 3A1c |
|  | Goats | EF (T2) | 3A1d |
|  | Camels | EF (T1) | 3A1e |
|  | Horses | EF (T1) | 3A1f |
|  | Mules | EF (T1) | 3A1gi |
|  | Donkeys | EF (T1) | 3A1gii |
|  | Swine | EF (T2) | 3A1h |
|  | Rabbit# | EF (T1) | 3A1j |
|  | Manure management* |  | 3A2 |
|  | Dairy Cows | EF (T2) | 3A2ai |
|  | Other Cattle | EF (T2) | 3A2aii |
|  | Buffalo | EF (T2) | 3A2b |
|  | Sheep | EF (T2) | 3A2c |
|  | Goats | EF (T2) | 3A2d |
|  | Camels | EF (T1) | 3A2e |
|  | Horses | EF (T1) | 3A2f |
|  | Mules | EF (T1) | 3A2gi |
|  | Donkeys | EF (T1) | 3A2gii |
|  | Swine | EF (T2) | 3A2h |
|  | Poultry | EF (T1) | 3A2i |
|  | Rabbit# | EF (T1) | 3A2j |
|  | Aggregate sources and non-CO_2_ emissions sources on land* |  | 3C |
|  | CH_4_ emissions from biomass burning* |  | 3C1 |
|  | Forest lands | EF | 3C1a |
|  | Croplands |  | 3C1b |
|  | Grasslands |  | 3C1c |
|  | Other land |  | 3C1d |
|  | Rice cultivation | PM | 3C7 |
|  | Aquaculture# | EF | 3C8 |
|  | Wetlands# | PM | 3C9 |
|  | Lakes# | DD | 3C10 |
|  | Reservoirs# | DD | 3C11 |
|  | Rivers and streams# | DD | 3C12 |
|  | Natural soil CH_4_ sink# | PM | 3C13 |
|  | Termites# | DD | 3C14 |
|  | Geological seepage# | DD | 3C15 |
| Waste | Solid waste disposal | EF (T2) | 4A |
|  | Wastewater treatment and discharge | EF (T1) | 4D |

^1^Method abbreviation: EF (emission factor method), PB (process-based model) and DD (data-driven method). ^2^Code indicates the corresponding code of category in the 2019 Refinement to the 2006 IPCC Guidelines on National Greenhouse Gas Inventories. *The emissions of these categories were not estimated directly, but the sum of their sub-categories. #These categories are newly added in this study not be included by in the 2019 Refinement to the 2006 IPCC Guidelines on National Greenhouse Gas Inventories.

Table S3. Sectors and categories of N_2_O sources included in China Greenhouse Gas emission dataset (CNGHG).

| **Sector** | **Source** | **Method^1^** | **Code^2^** |
| --- | --- | --- | --- |
| Energy | Fuel combustion activities* |  | 1A |
|  | Energy industries* |  | 1A1 |
|  | Main activity electricity and heat production* |  | 1A1a |
|  | Electricity Generation | EF (T1) | 1A1ai |
|  | Heat Plants |  | 1A2aiii |
|  | Petroleum Refining | EF (T1) | 1A1b |
|  | Manufacture of solid fuels and other energy industries* |  | 1A1c |
|  | Manufacture of Solid Fuels | EF (T1) | 1A1ci |
|  | Gas Production |  | 1A1cii |
|  | Manufacturing Industries & Construction | EF (T1) | 1A2 |
|  | Transport | EF (T2) | 1A3 |
|  | Other sectors* |  | 1A4 |
|  | Residential | EF (T1) | 1A4b |
|  | Agriculture/Forestry/Fishing/Fish Farms |  | 1A4c |
|  | Non-Specified | EF (T1) | 1A5 |
|  | Fugitive emissions from fuels* |  | 1B |
|  | Oil and natural gas |  | 1B2 |
| Industrial processes and product use | Chemical industry* |  | 2B |
|  | Nitric acid production | EF (T2) | 2B2 |
|  | Adipic acid production |  | 2B3 |
| Agriculture, forestry, and other land use | Livestock* |  | 3A |
|  | Manure management | EF (T2, T1) | 3A2 |
|  | Natural soils | PM, DD | 3B |
|  | Aggregate sources and non-CO_2_ emissions sources on land* |  | 3C |
|  | Emissions from biomass burning* |  | 3C1 |
|  | Emissions from biomass burning in forest lands | EF | 3C1a |
|  | Emissions from biomass burning in croplands |  | 3C1b |
|  | Emissions from biomass burning in grasslands |  | 3C1c |
|  | Emissions from biomass burning in other lands |  | 3C1d |
|  | Direct N_2_O emissions from managed soils* |  | 3C4 |
|  | Fertilizer and manure application in cropland | DD | 3C4a |
|  | N mineralization in cropland |  | 3C4b |
|  | Crop residue in cropland |  | 3C4c |
|  | Nitrogen inputs from fertilizer in pasture |  | 3C4d |
|  | Indirect N_2_O emissions from manure management* |  | 3C5 |
|  | Nitrogen deposition in cropland | DD | 3C5a |
|  | Nitrogen leaching in cropland | EF (T1) | 3C5b |
|  | Aquaculture# | EF | 3C8 |
|  | Lakes# | DD | 3C10 |
|  | Reservoirs# | DD | 3C11 |
|  | Rivers and streams# | DD | 3C12 |
| Waste | Wastewater treatment and discharge | EF (T2) | 4D |

^1^Method abbreviation: EF (emission factor method), PB (process-based model) and DD (data-driven method). ^2^Code indicates the corresponding code of category in the 2019 Refinement to the 2006 IPCC Guidelines on National Greenhouse Gas Inventories. *The emissions of these categories were not estimated directly, but the sum of their sub-categories. #These categories are newly added in this study not be included by in the 2019 Refinement to the 2006 IPCC Guidelines on National Greenhouse Gas Inventories.

**Table S4.** The national GHG budget for three greenhouse gases averaged from 2012 to 2021.

| **Sectors** | **Categories** | **CO_2_**  **(Tg CO_2_ yr^-1^)** | **CH_4_**  **(Tg CH_4_ yr^-1^)** | **N_2_O**  **(Gg N_2_O yr^-1^)** |
| --- | --- | --- | --- | --- |
| **Energy** |  | **9311.50** | **25.57** | **317.19** |
|  | Power industry | 4485.11 |  | 238.09 |
|  | Oil refineries and transformation industry | 295.26 |  | 10.35 |
|  | Combustion for manufacturing | 3015.29 |  | 39.89 |
|  | Transport | 642.28 |  | 21.07 |
|  | Energy for buildings | 779.13 |  | 7.76 |
|  | Fuel exploitation | 94.43 |  | 0.03 |
|  | Fuel combustion activities |  | 1.55 |  |
|  | Solid fuels |  | 21.89 |  |
|  | Oil and natural gas |  | 2.13 |  |
| **IPPU (industrial processes and product use)** |  | **1204.98** |  | **388.73** |
|  | Cement production | 768.66 |  |  |
|  | Chemical industry | 280.18 |  |  |
|  | Metal industry | 156.14 |  |  |
|  | Nitric acid production |  |  | 39.25 |
|  | Adipic acid production |  |  | 349.49 |
| **AFOLU (agriculture, forestry and other land use)** |  | **-1048.65** | **29.21** | **1616.84** |
|  | NBP | -1189.03 |  |  |
|  | Natural soil |  | -2.35 | 561.31 |
|  | Wetlands |  | 1.29 |  |
|  | Biomass burning | 79.59 | 0.28 | 6.43 |
|  | Lakes | 32.80 | 3.27 | 37.62 |
|  | Reservoirs | 27.99 | 1.19 | 20.66 |
|  | Enteric fermentation |  | 10.50 |  |
|  | Manure management |  | 3.14 | 320.78 |
|  | Agricultural Soil |  | 8.66 | 646.18 |
|  | Aquaculture |  | 3.22 | 23.87 |
| **Waste** |  |  | **5.88** | **116.94** |
|  | Solid waste disposal |  | 3.59 |  |
|  | Wastewater treatment and discharge |  | 2.29 | 116.94 |
| **Bottom-up Net GHG emission** | | **9467.83** | **60.66** | **2439.71** |
| **Bottom-up Gross GHG emission** | | **10656.86** | **63.01** | **2439.71** |

Table S5. Set up and input datasets for atmospheric inversions.

|  | GCASv2 | GONGGA v2023 | GONGGA-CN v2023 |
| --- | --- | --- | --- |
| Period covered | 2015.1-2022.12 | | |
| Horizontal Resolution | 1.875°×2.5° | 2°×2.5° | ~ 50 km |
| Atmospheric observations | OCO-2 ACOS v11.1 | OCO-2 ACOS v11.1r | OCO-2 ACOS v11.1r |
| Fossil fuels | China: MEIC before 2020; After 2020, the emissions of MEIC 2020 were scaled to Carbon Monitor’s estimate of annual budget; Rest of World: GridFEDv2023.1 | | |
| Prior biosphere and fire fluxes | BEPS  GFEDv4.1s | ORCHIDEE-MICT  GFEDv4.1s | ORCHIDEE-MICT  GFEDv4.1s |
| Prior ocean-air exchanges | JMA_co2map_v2023 | JMA_co2map_v2023 | Takahashi Climatology |
| Transport model | MOZART-4 | GEOSChem v12.9.3 | WRF v4.3  CMAQ v5.3 |
| Weather forcing | GEOS-5 | MERRA2 | ERA5 |
| Optimization Method | EnSRF | NLS-4DVar | NLS-4DVar |

Table S6. Set up and input fields for atmospheric inversion of CH_4_ emissions.

|  | Westlake | GONGGA-CH4 v2023 |
| --- | --- | --- |
| Period covered | 2019.1-2022.12 | 2010.1-2022.12 |
| Horizontal Resolution | 0.5°×0.625° | 2°×2.5° |
| Atmospheric observations | University of Leicester GOSAT Proxy XCH4 V9.0;  or blended TROPOMI + GOSAT data (Balasus et al., AMT, 2023); | University of Leicester GOSAT proxy XCH_4_ v9.0 |
| Anthropogenic sources | EDGAR v7.0 | EDGAR v6.0; or EDGAR v7.0 |
| Wetland | VISIT | VISIT v20230209b |
| Open fires | GFAS | GFED4 |
| Other natural sources | freshwater, termites, ocean, geological:  Saunois et al. (2024) ^[47]^ | CarbonTracker-Europe |
| Transport model | GEOS-Chem v12.9.3 | GEOS-Chem v14.2.1 |
| Weather forcing | MERRA2 | MERRA2 |
| Optimization Method | Analytical Bayesian optimization | NLS-4DVar |

Table S7. Statistics yearbooks used in this study for various sectors.

| **Sector** | **Code^1^** | **Yearbook** |
| --- | --- | --- |
| Energy | 1A, 1B | China energy statistical yearbook |
| Industrial processes and product use | 2A | China industry statistical yearbook |
|  | 2B | China chemical industry yearbook |
|  | 2C1 | China steel yearbook |
| Agriculture, forestry, and other land use | 3A | China animal husbandry and veterinary yearbook |
| Waste | 4A, 4D | China statistical yearbook on environment |

^1^Code indicates the categories of Table S1-S3.

Table S8. Sectoral comparisons between our estimates (China Greenhouse Gas Dataset, CNGHG) and National Greenhouse Gas Inventory (NGHGI) averaged over five years (2010, 2012, 2014, 2017 and 2018).

| **Sector^1^** | **CO_2_**  (Million-ton CO_2_ yr^-1^) | | | **CH_4_**  (Million-ton CH_4_ yr^-1^) | | | **N_2_O**  (Million-ton N_2_O yr^-1^) | | |
| --- | --- | --- | --- | --- | --- | --- | --- | --- | --- |
|  | **CNGHG** | **NGHGIs** | **Diff** | **CNGHG** | **NGHGIs** | **Diff** | **CNGHG** | **NGHGIs** | **Diff** |
| **Total (including LULUCF)** | 887824.5 | 900425.9 | -1.4% | 5437.1 | 5909.0 | -8.0% | 166.4 | 192.4 | -13.5% |
| **Energy activities** | 888959.0 | 878685.8 | 1.2% | 2508.2 |  | -8.1% | 30.4 | 33.7 | -9.8% |
| Fuel combustion | 877223.5 | 878685.8 | -0.2% | 150.1 | 233.4 |  | 30.4 | 33.7 | -9.8% |
| Energy industry | 439915.4 | 401791.2 | 9.5% |  | 6.7 |  | 23.3 | 20.6 | 13.1% |
| Manufacturing Industries and construction | 301955.4 | 324897.6 | -7.1% |  | 28.1 |  | 4.3 | 6.3 | -32.7% |
| Transport | 60338.3 | 84170.0 | -28.3% |  | 9.8 |  | 2.0 | 2.1 | -4.6% |
| Other sectors | 75014.3 | 63828.9 | 17.5% |  | 94.4 |  | 0.8 | 1.3 | -36.6% |
| Fugitive emissions from fuels |  |  |  | 2358.1 | 2496.9 | -5.6% |  |  |  |
| **Industrial production processes** | 112940.0 | 129584.9 | -12.8% |  | 0.5 |  | 30.6 | 32.8 | -6.9% |
| Non-metallic minerals production | 72717.4 | 89214.2 | -18.5% |  |  |  |  |  |  |
| Chemical industry | 25792.2 | 19129.0 | 34.8% |  |  |  | 30.6 | 32.8 | -6.9% |
| **Agricultural activities** |  |  |  | 2258.1 | 2322.0 | -2.8% | 97.3 | 114.5 | -15.0% |
| Enteric fermentation |  |  |  | 1049.2 | 1057.8 | -0.8% |  |  |  |
| Manure management |  |  |  | 322.2 | 330.9 | -2.6% | 32.4 | 23.1 | 40.4% |
| Rice cultivation |  |  |  | 854.5 | 901.1 | -5.2% |  |  |  |
| Agricultural soils |  |  |  |  |  |  | 64.2 | 90.6 | -29.2% |
| Agricultural waste burning |  |  |  | 32.3 | 32.2 | 0.3% | 0.7 | 0.8 | -7.7% |
| **Land use, land-use change and forestry** | -115051.5 | -108773.3 | 5.8% | 127.8 | 228.8 | -44.2% |  | 0.1 |  |
| **Waste** |  | 928.4 |  | 543.0 | 627.4 | -13.5% | 8.1 | 11.3 | -28.5% |
| Solid waste |  | 928.4 |  | 316.0 | 355.0 | -11.0% |  | 0.7 |  |
| Waste water |  |  |  | 226.9 | 272.4 | -16.7% | 8.1 | 10.6 | -23.9% |

^1^Sector category is based on national greenhouse gas inventory. This table did not include 2005 as updated national greenhouse gas inventory at 2005 only provided the total emission without sectoral emissions.

Table S9. Emission sources should be included by the statistical system.

| **Sector** | **Source and gas** | **Variable** |
| --- | --- | --- |
| Energy | Transport (CO_2_ and N_2_O) | Magnitude of fossil fuels for various transportation mode |
| Industrial processes and product use | Cement production (CO_2_) | Clinker production |
|  | Metal production (CO_2_) | Consumption of dolomite and limestone |
|  | Chemical industry (CO_2_) | Production of dichloroethane, oxirane, acrylonitrile and black carbon; Consumption of titanium slag and rutile |
|  | Adipic acid (N_2_O) | Production and mitigation measures |
|  | Nitric acid (N_2_O) | Production and mitigation measures |
| Agriculture, forestry and other land use | Manure management (CH_4_ and N_2_O) | Fraction of each management measure |
|  | Crop residue in cropland (CO_2_ and N_2_O) | Fraction of straw returning in cropland |
|  | Rice cultivation (CH_4_) | Water management |
| Waste | Wastewater treatment and discharge (CH_4_ and N_2_O) | Technologies of wastewater treatment and discharge |
|  | Biological treatment of solid waste & Waste incineration (CO_2_, CH_4_ and N_2_O) | Fraction of residual waste and household food waste |

Table S10. Categories of food and wood trade and carbon content.

| **Category** | **Content (%)** | **Reference** |
| --- | --- | --- |
| Wine | 0.180 | USDA, Agricultural Research Service, 2022 ^[48]^ |
| Beer | 0.019 | USDA, Agricultural Research Service, 2022 |
| Animal fats | 0.771 | USDA, Agricultural Research Service, 2022 |
| Aquatic Products, Other | 0.125 | USDA, Agricultural Research Service, 2022 |
| Cereals - Excluding Beer | 0.408 | Ciais et al., 2007 ^[49]^ |
| Eggs | 0.148 | USDA, Agricultural Research Service, 2022 |
| Fish, Seafood | 0.139 | USDA, Agricultural Research Service, 2022 |
| Fruits - Excluding Wine | 0.05 | Baes et al., 1984 ^[50]^ |
| Bovine meat | 0.147 | USDA, Agricultural Research Service, 2022 |
| Poultry meat | 0.181 | USDA, Agricultural Research Service, 2022 |
| Pigmeat | 0.365 | USDA, Agricultural Research Service, 2022 |
| Mutton & Goat Meat | 0.218 | USDA, Agricultural Research Service, 2022 |
| Milk - Excluding Butter | 0.063 | USDA, Agricultural Research Service, 2022 |
| Offals | 0.154 | USDA, Agricultural Research Service, 2022 |
| Oilcrops | 0.54 | Ciais et al., 2007 |
| Pulses | 0.45 | Ciais et al., 2007 |
| Spices | 0.045 | Ciais et al., 2007 |
| Starchy Roots | 0.11 | Ciais et al., 2007 |
| Sugar & Sweeteners | 0.4 | USDA, Agricultural Research Service, 2022 |
| Sugar Crops | 0.085 | Ciais et al., 2007 |
| Treenuts | 0.57 | Ciais et al., 2007 |
| Vegetable Oils | 0.54 | Ciais et al., 2007 |
| Vegetables | 0.026 | Baes et al., 1984 |


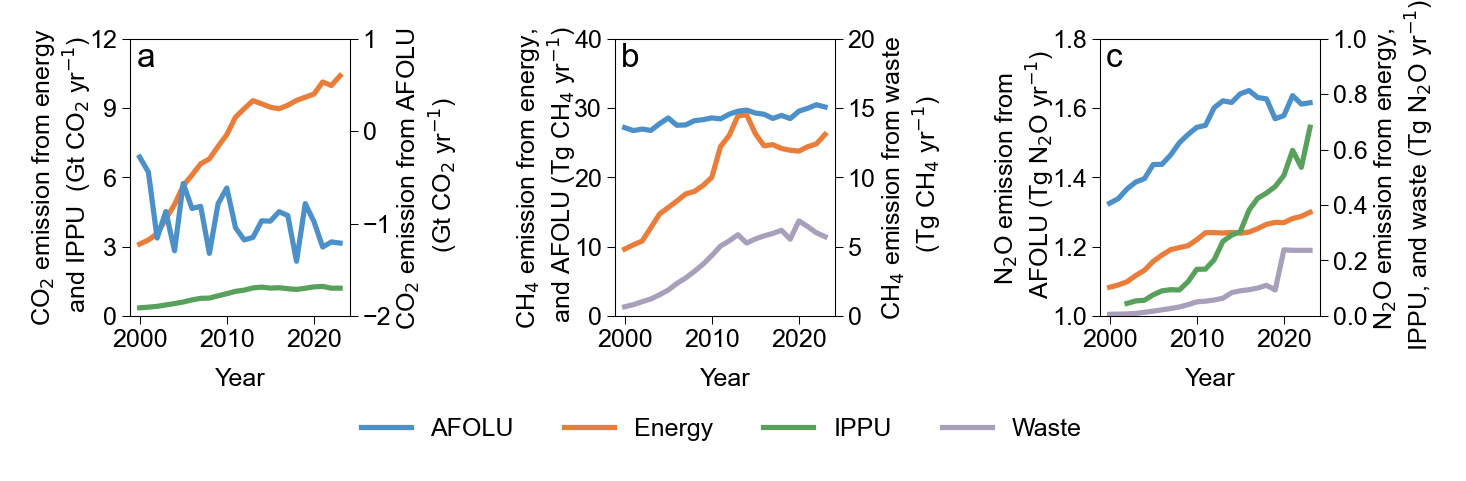


Figure S1. Long-term variations of CO_2_ (a), CH_4_ (b) and N_2_O (c) emissions and sinks over four sectors through 2000-2023. IPPU indicates the industrial processes and product use sector, and AFOLU indicates agriculture, forestry, and other land use sector.


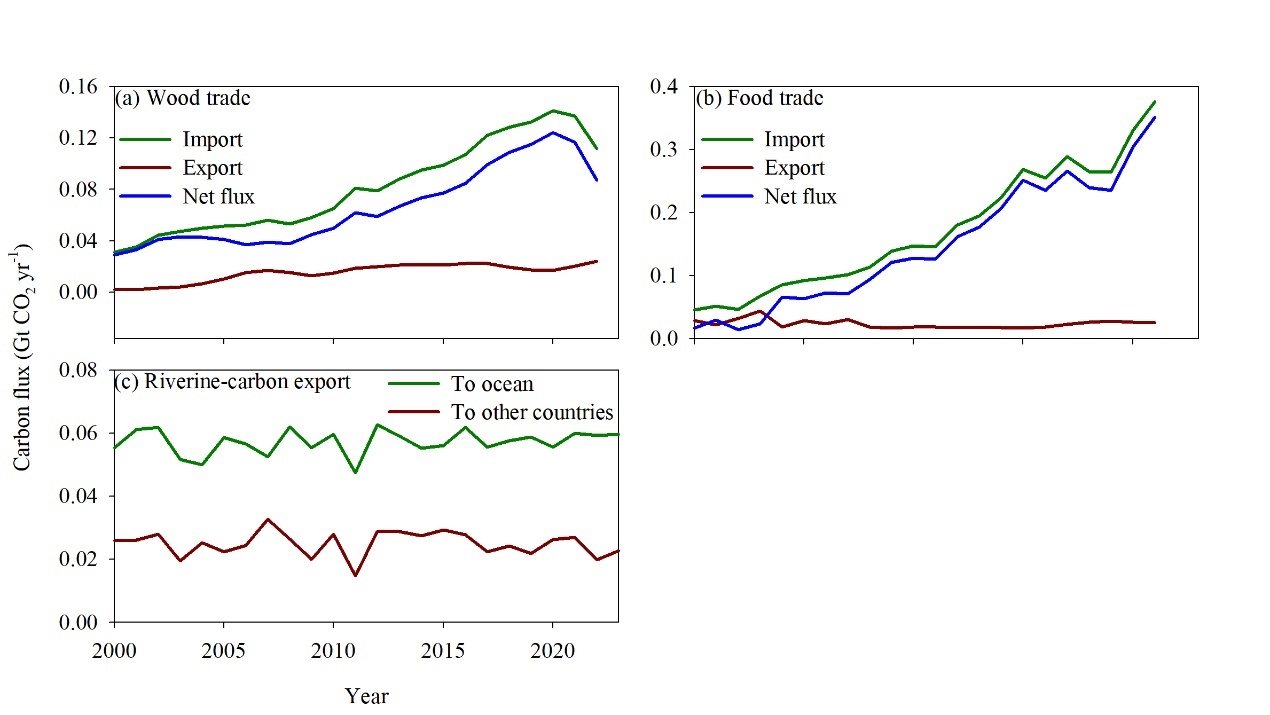
Figure S2. Long-term variations of lateral carbon fluxes through 2000-2023. (a) wood trade, (b) food trade and (c) riverine-carbon export.


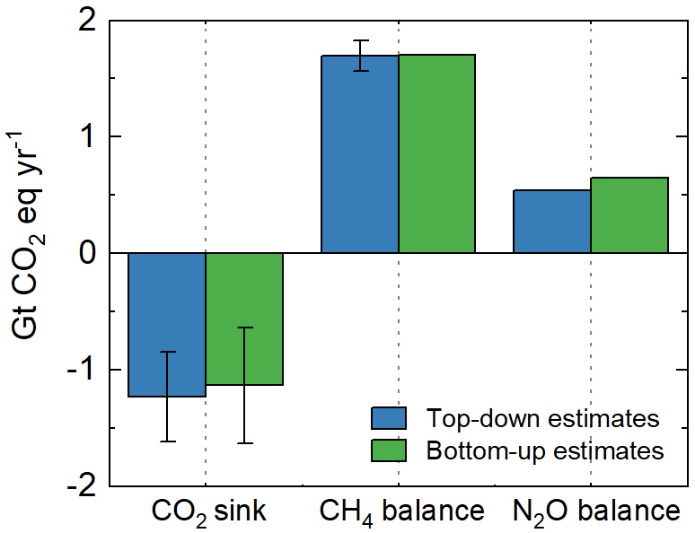
Figure S3. Comparisons of GHG estimates from bottom-up and top-down approaches. Top-down estimates of CO_2_ sink is from 3 inversions (GONGGA, GONGGA-CN, GCASv2), and bottom-up estimates of it come from 6 process-based ecosystem models (BEPS, IBIS, LPJ-GUESS, ORCHIDEE, TRIPLEX-GHG, iMAPLE). The estimates were adjusted for lateral fluxes during 2015-2022. The CH_4_ balance is estimated from 2 inversions (Westlake and GONGGA, see Table S6) and the inventory-based bottom-up estimates across all sectors between 2011 and 2022. The N_2_O balance is derived from the PKU inversion system and the inventory-based estimates spanning 2009 to 2022. Error bars are presented only when multiple sets of estimates are available. Details of the data products used can be found from Table S1-S6 and Text S1.5.


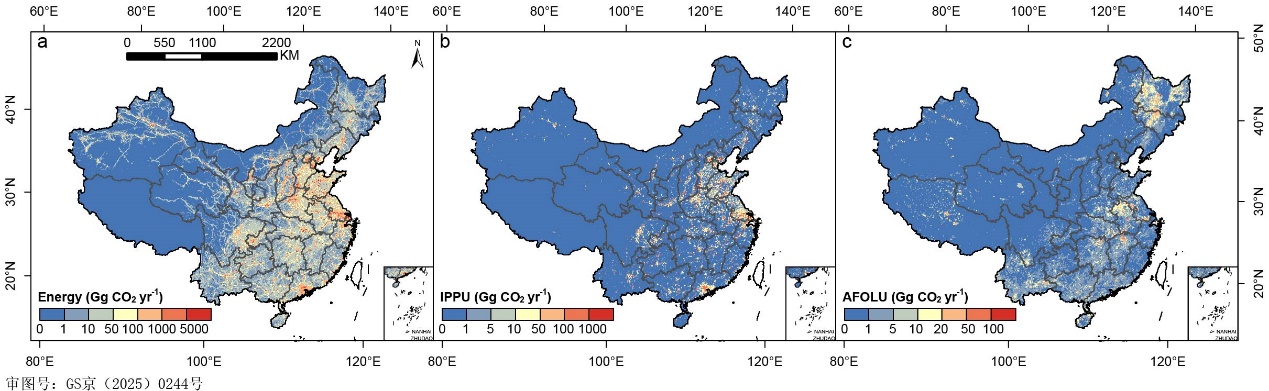


**Figure S4**. Spatial distribution of gross CO_2_ emissions from the energy (a), industrial processes and product use (IPPU, b), and agriculture, forestry, and other land use (AFOLU, c) sectors in China averaged through 2012–2021. The values represent emissions for each 100 km^2^ grid cell. Data for Taiwan province is not included.


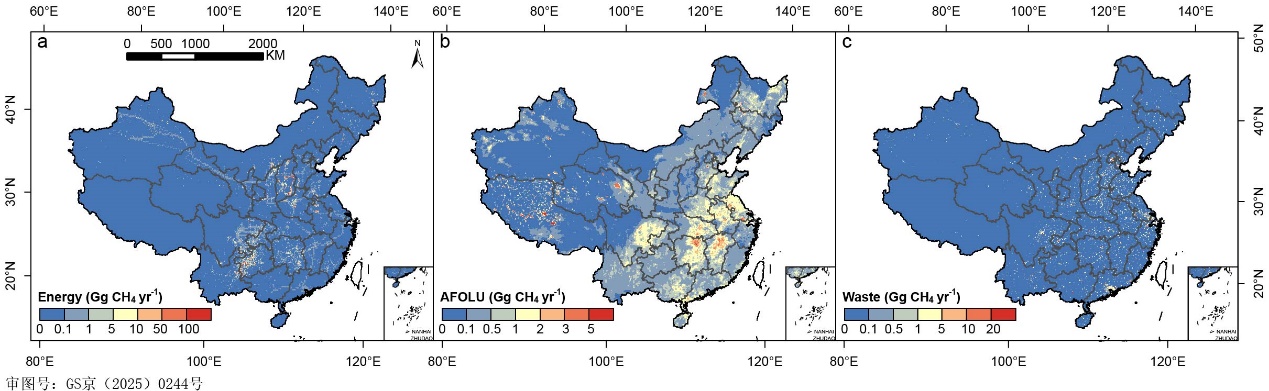


**Figure S5**. Spatial distribution of gross CH_4_ emissions from the energy (a), agriculture, forestry, and other land use (AFOLU, b) and waste (c) sectors in China averaged through 2012–2021. The values represent emissions for each 100 km^2^ grid cell. Data for Taiwan province is not included.


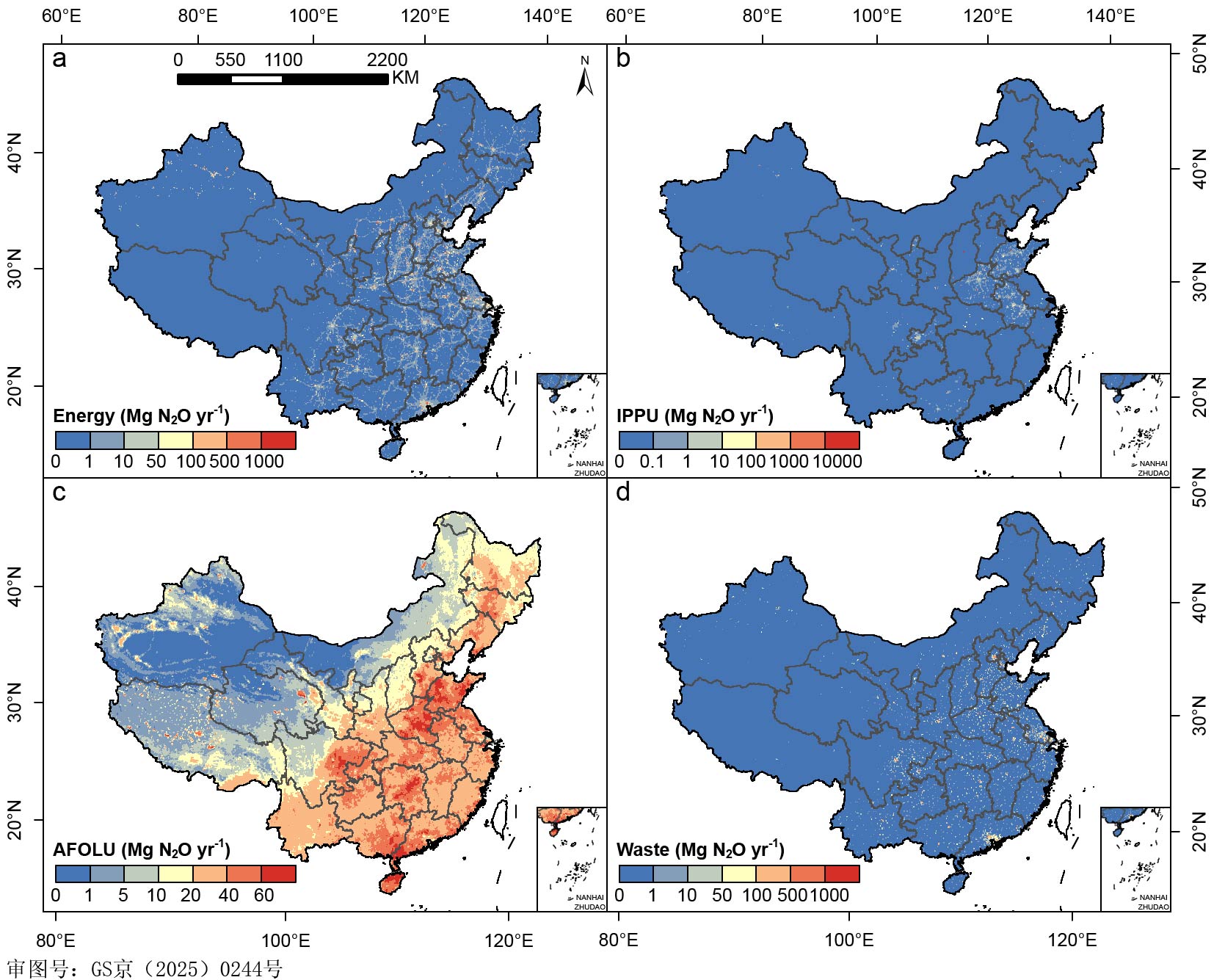


**Figure S6**. Spatial distribution of gross N_2_O emissions from the energy (a), industrial processes and product use (IPPU, b), agriculture, forestry, and other land use (AFOLU, c) and waste (d) sectors in China averaged through 2012–2021. The values represent emissions for each 100 km^2^ grid cell. Data for Taiwan province is not included.


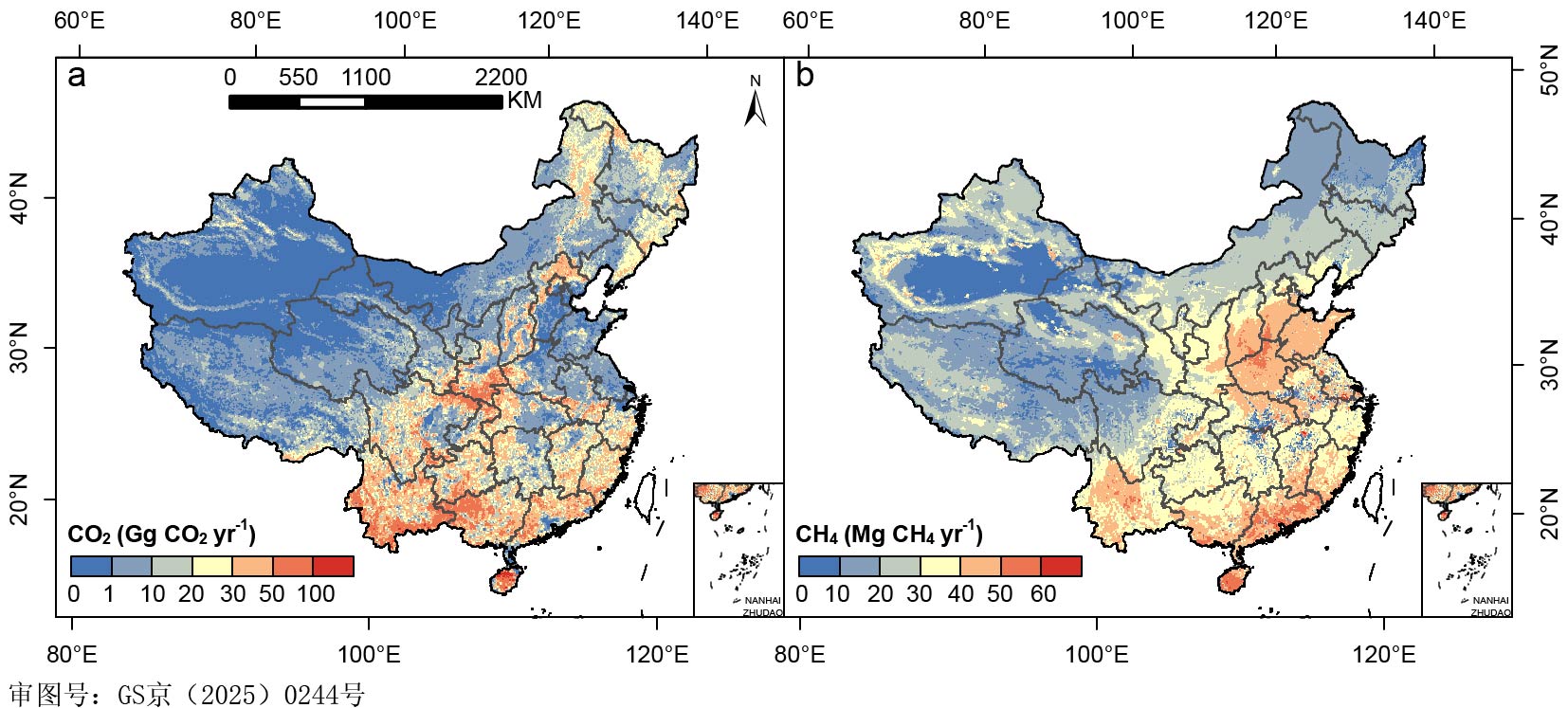


Figure S7. Spatial pattern of terrestrial CO_2_ (a) and CH_4_ (b) sinks. The values are averaged from 2012 to 2021 and represent emissions for each 100 km^2^ grid cell. Data for Taiwan province is not included.


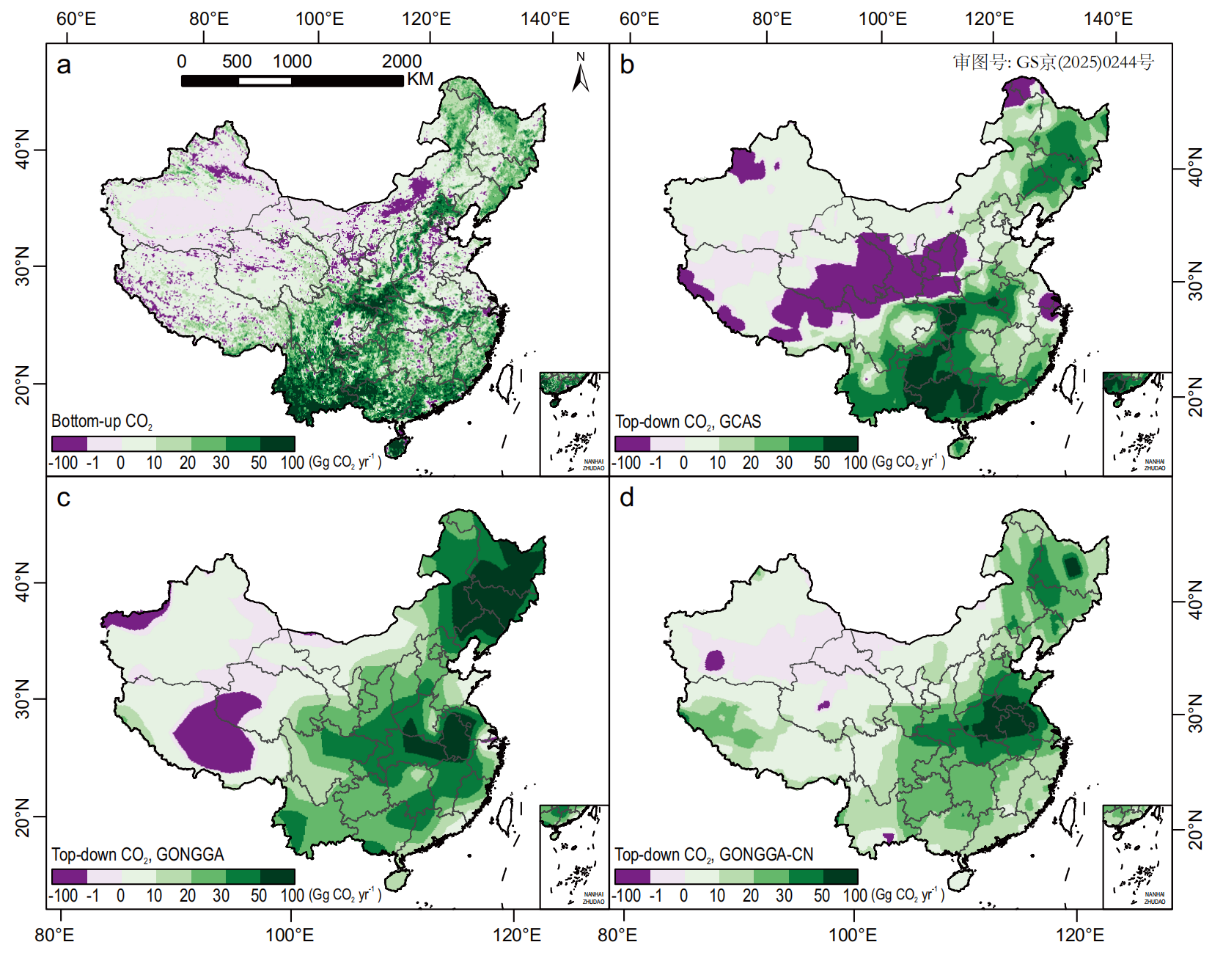


Figure S8. Spatial pattern of terrestrial CO_2_ sinks based on bottom-up and top-down methods, averaged from 2015 to 2022. Bottom-up estimates are derived from six process-based ecosystem models (a). Top-down terrestrial CO_2_ sink estimates are provided by (b) GCASv2, (c) GONGGA, and (d) GONGGA-CN inversions. The values represent CO_2_ sinks for each 100 km^2^ grid cell. Negative values indicate CO_2_ emissions. Data for Taiwan province is not included.


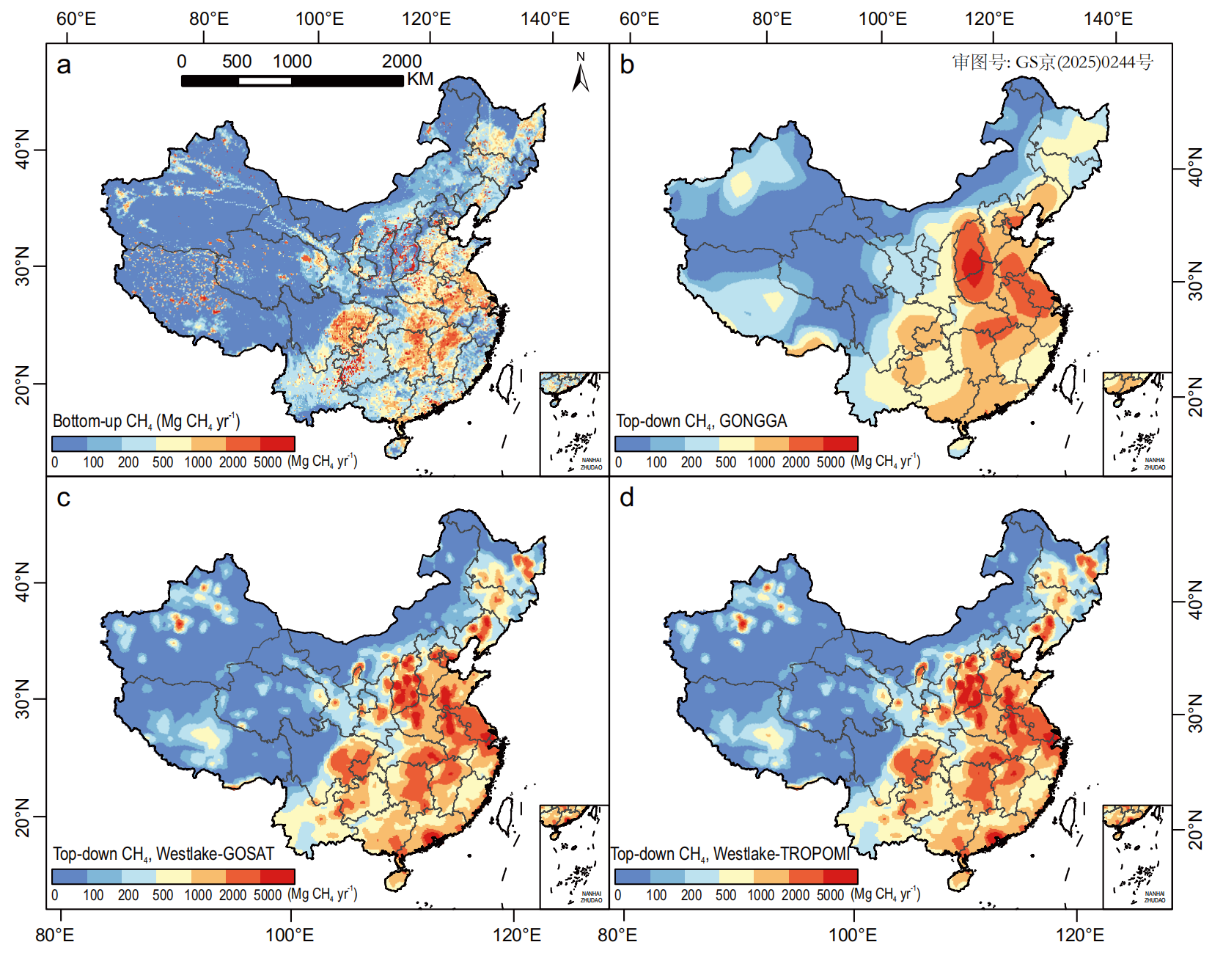


Figure S9. Spatial pattern of net CH_4_ emissions based on bottom-up and top-down methods, averaged from 2019 to 2022. (a) Bottom-up estimates, (b) GONGGA, (c) Westlake-GOSAT, and (d) Westlake-TROPOMI. The values represent emissions for each 100 km^2^ grid cell. Data for Taiwan province is not included.


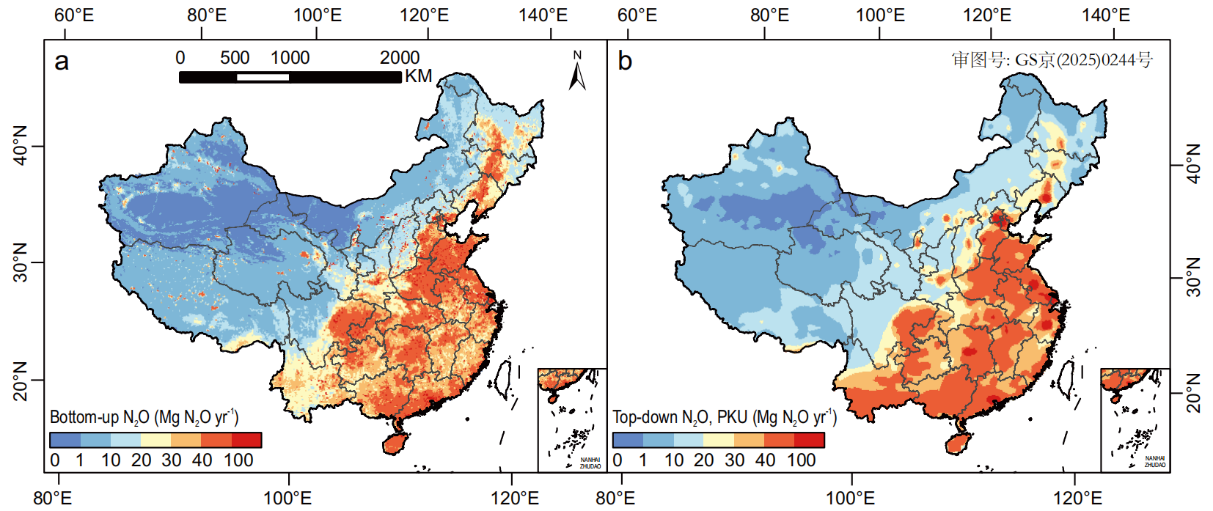


Figure S10. Spatial pattern of net N_2_O emissions based on bottom-up (a) and PKU inversion system (b), averaged from 2009 to 2022. The values represent emissions for each 100 km^2^ grid cell. Due to observational limitations, the atmospheric inversion estimates for western China shown here are based on prior fluxes. Data for Taiwan province is not included.


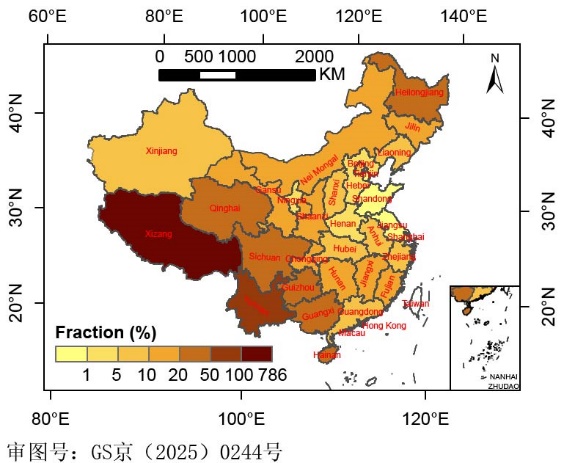


Figure S11. Fraction between terrestrial carbon sink and CO_2_ emission from energy and industry sectors. The values are mean from 2012 to 2021. Data for Taiwan province is not included.





Figure S12. Long-term changes of energy consumption (a) and cement production (b) during 2000-2023.


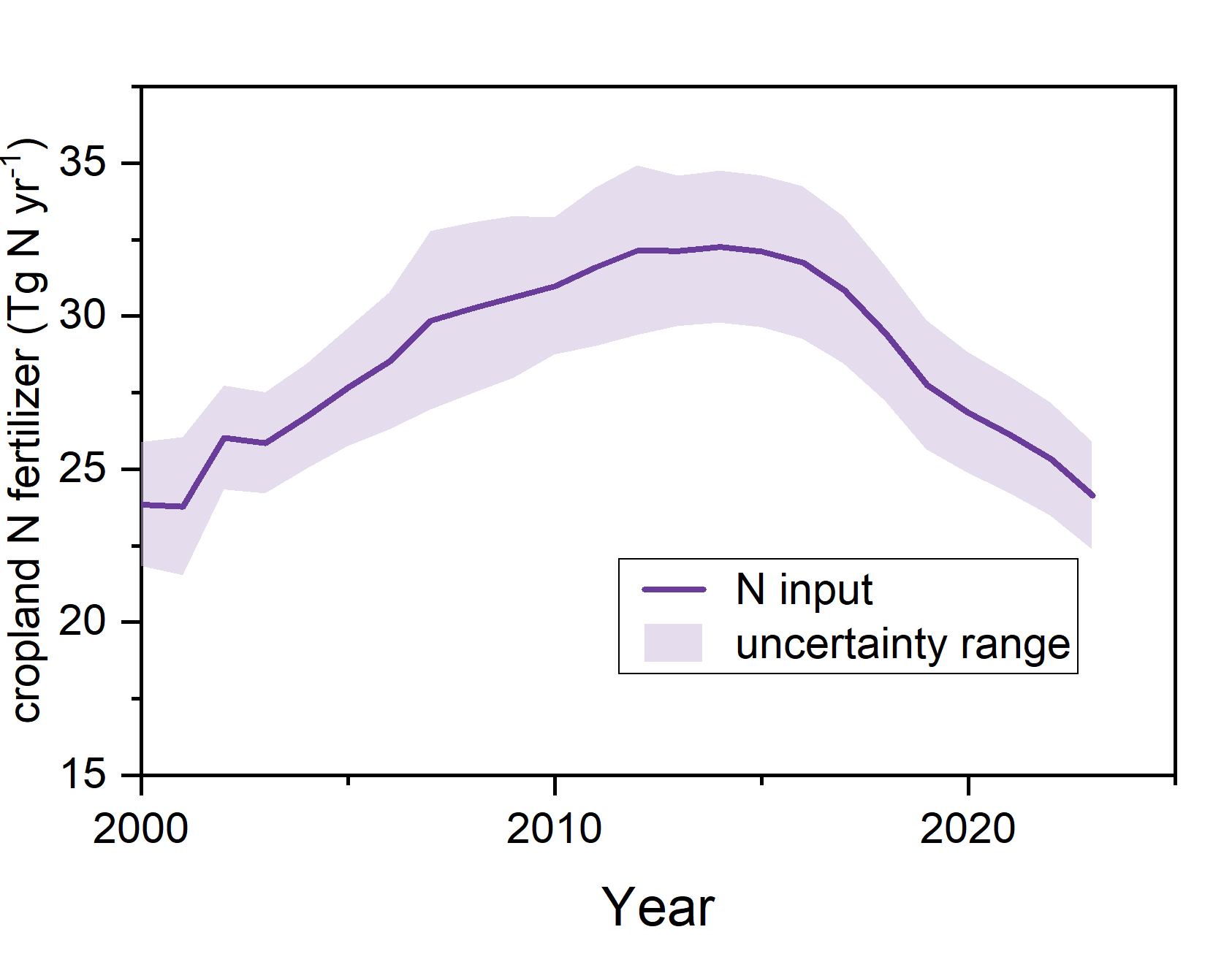


Figure S13. Long-term change of nitrogen fertilizer application in cropland during 2000-2023. Detailed information on nitrogen fertilizer data can be found in the reference ^[25]^.


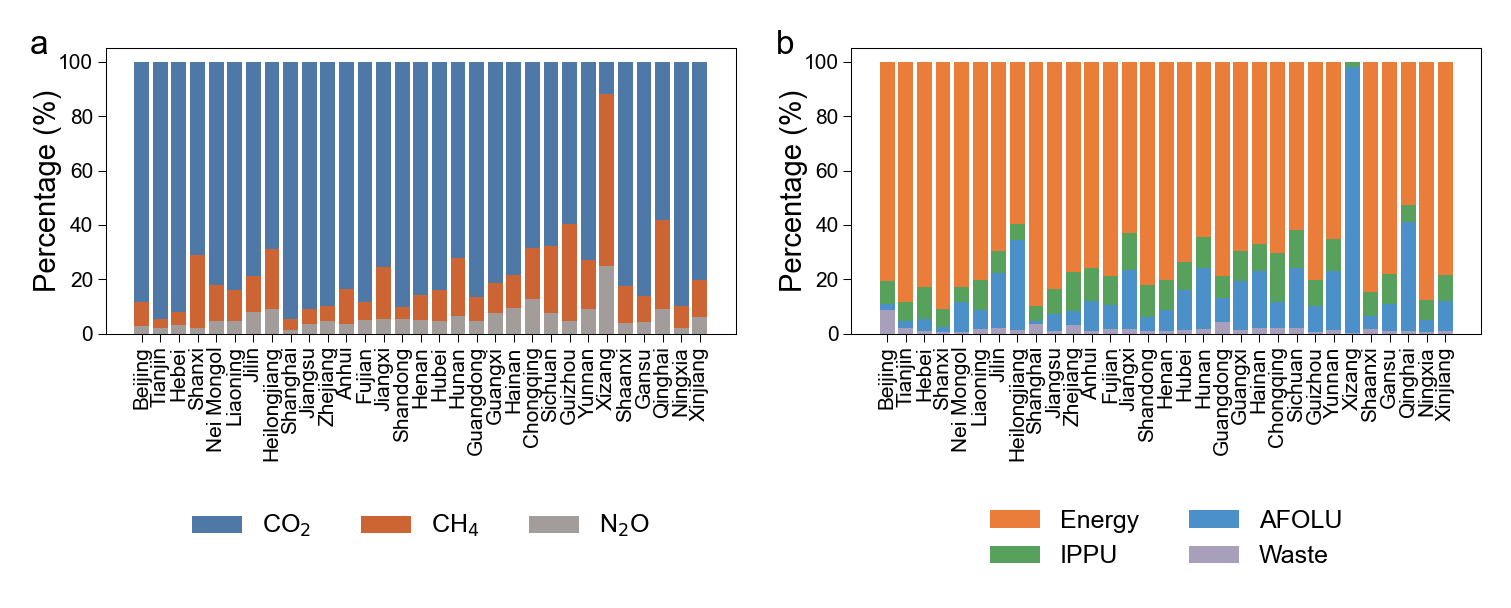


Figure S14. Percentage of emissions among three greenhouse gases (a) and four sectors (b) for each province. The values were the mean from 2012 to 2021.


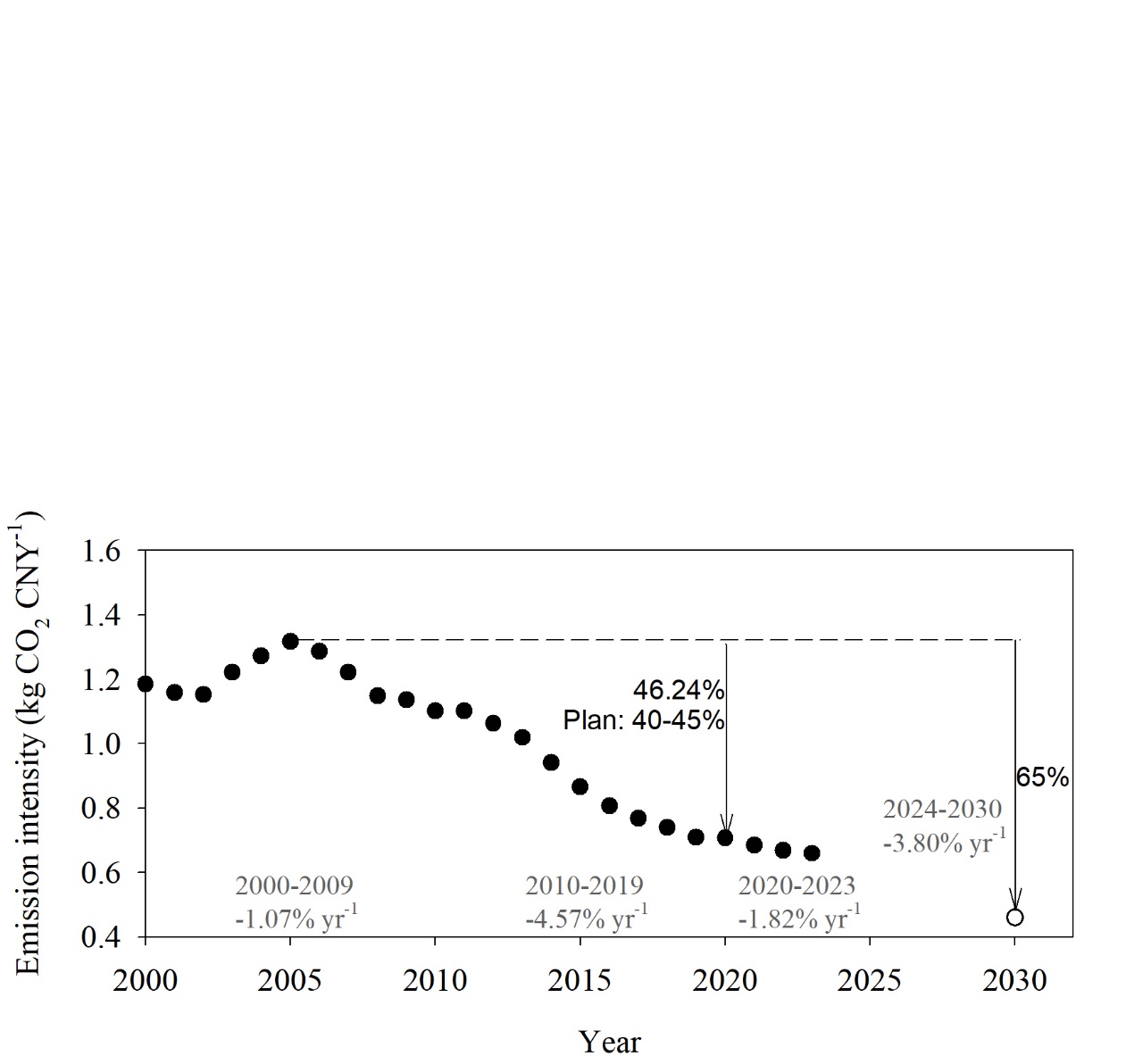


**Figure S15**. Temporal variation of emission intensity from 2000 to 2023 in China. The emission intensity is defined as the anthropogenic CO_2_ emission per gross domestic product.


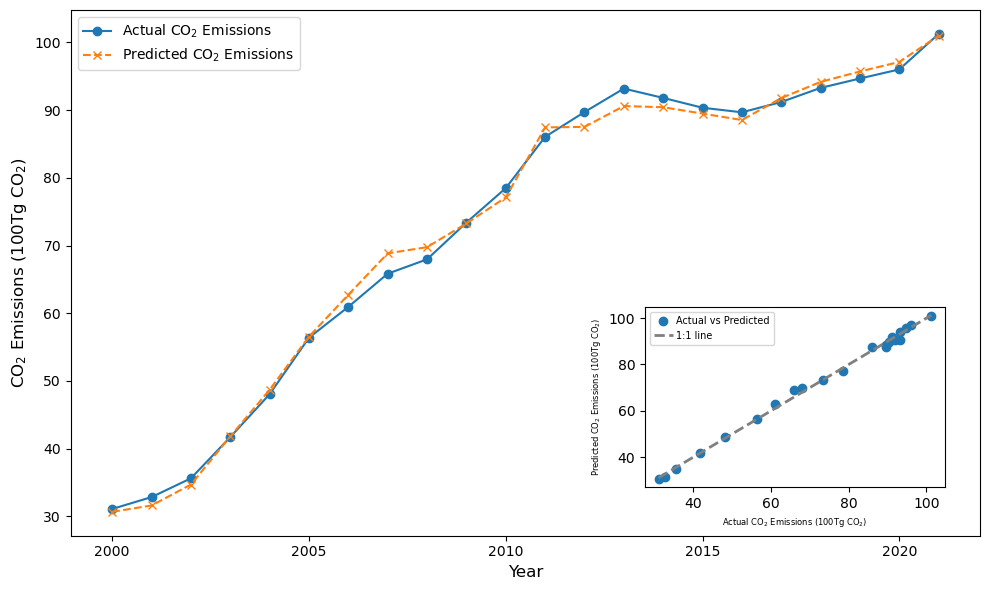


Figure S16. The validation of multiple linear regression equation for estimating CO_2_ emissions of entire energy sector.

Reference

1. IPCC. 2019 Refinement to the 2006 IPCC Guidelines for National Greenhouse Gas Inventories. Hyama: Institute for Global Environmental Strategies; 2019.

2. China National Development and Reform Commission. Guidelines for provincial greenhouse gas inventories (in Chinese). 2011. Available at <http://www.edcmep.org.cn/tzh/ptfb/zcbz/202106/P020210601177040314696.pdf>

3. Liu Q, Teng F, Nielsen CP *et al.* Large methane mitigation potential through prioritized closure of gas-rich coal mines. *Nature Climate Change*. 2024; **14**(6): 652-658.

4. Kholod N, Evans M, Pilcher RC *et al.* Global methane emissions from coal mining to continue growing even with declining coal production. *Journal of Cleaner Production*. 2020; **256**: 120489.

5. Gan Y, El-Houjeiri HM, Badahdah A *et al.* Carbon footprint of global natural gas supplies to China. *Nature Communications*. 2020; **11**(1): 824.

6. Masnadi MS, El-Houjeiri HM, Schunack D *et al.* Well-to-refinery emissions and net-energy analysis of China’s crude-oil supply. *Nature Energy*. 2018; **3**(3): 220-226.

7. China National Development and Reform Commission. The People’s Republic of China First Biennial Update Report on Climate Change. 2016. Available at <https://www.mee.gov.cn/ywgz/ydqhbh/wsqtkz/201904/P020190419522735276116.pdf>

8. China National Development and Reform Commission. The People’s Republic of China Second Biennial Update Report on Climate Change. 2018. Available at http://tnc.ccchina.org.cn/archiver/NCCCcn/UpFile/Files/Htmleditor/202007/20200723155226725.pdf

9. Jing C, Su B, Zhai J *et al.* Gridded value-added of primary, secondary and tertiary industries in China under Shard Socioeconomic Pathways. *Scientific Data*. 2022; **9**(1): 309.

10. Liang M, Zhou Z, Ren P *et al.* Four decades of full-scale nitrous oxide emission inventory in China. *National Science Review*. 2024: nwad285.

11. Tkachenko N, Tang K, McCarten M *et al.* Global database of cement production assets and upstream suppliers. *Scientific Data*. 2023; **10**(1): 696.

12. Huaon. Report of Market Development Monitoring and Investment Strategy Planning Research on China adipic acid Industry (in Chinese). 2023.

13. Huaon. Report of Market Development Monitoring and Investment Strategy Planning Research on China nitric acid Industry (in Chinese). 2023.

14. Chen J, Ju W, Ciais P *et al.* Vegetation structural change since 1981 significantly enhanced the terrestrial carbon sink. *Nature Communications*. 2019; **10**(1): 4259.

15. Lu H, Yuan W, Chen X. A Processes-Based Dynamic Root Growth Model Integrated Into the Ecosystem Model. *Journal of Advances in Modeling Earth Systems*. 2019; **11**(12): 4614-4628.

16. Smith B, Wårlind D, Arneth A *et al.* Implications of incorporating N cycling and N limitations on primary production in an individual-based dynamic vegetation model. *Biogeosciences*. 2014; **11**(7): 2027-2054.

17. Krinner G, Viovy N, de Noblet-Ducoudré N *et al.* A dynamic global vegetation model for studies of the coupled atmosphere-biosphere system. *Global Biogeochemical Cycles*. 2005; **19**(1).

18. Zhu Q, Liu J, Peng C *et al.* Modelling methane emissions from natural wetlands by development and application of the TRIPLEX-GHG model. *Geoscientific Model Development*. 2014; **7**(3): 981-999.

19. Yue X, Unger N. The Yale Interactive terrestrial Biosphere model version 1.0: description, evaluation and implementation into NASA GISS ModelE2. *Geoscientific Model Development*. 2015; **8**(8): 2399-2417.

20. Qin Z, Zhu Y, Canadell JG *et al.* Global spatially explicit carbon emissions from land-use change over the past six decades (1961–2020). *One Earth*. 2024; **7**(5): 835-847.

21. Xia X, Ren P, Wang X *et al.* The carbon budget of China: 1980–2021. *Science Bulletin*. 2024; **69**(1): 114-124.

22. Zhang Q, Li T-T, Zhang Q *et al.* Accuracy analysis in CH4MOD_wetland_ in the simulation of CH4 emissions from Chinese wetlands. *Advances in Climate Change Research*. 2020; **11**(1): 52-59.

23. Song C, Luan J, Xu X *et al.* A Microbial Functional Group-Based CH4 Model Integrated Into a Terrestrial Ecosystem Model: Model Structure, Site-Level Evaluation, and Sensitivity Analysis. *Journal of Advances in Modeling Earth Systems*. 2020; **12**(4): e2019MS001867.

24. Li T, Canadell JG, Yang X-Q *et al.* Methane Emissions from Wetlands in China and Their Climate Feedbacks in the 21st Century. *Environmental Science & Technology*. 2022; **56**(17): 12024-12035.

25. Gao Y *et al*. Recent stabilization of agricultural non-CO2 greenhouse gas emissions in China. *Under review in this issue.*

26. Zhou M, Butterbach-Bahl K. Assessment of nitrate leaching loss on a yield-scaled basis from maize and wheat cropping systems. *Plant and Soil*. 2014; **374**(1): 977-991.

27. Huang Y, Zhang W, Zheng X *et al.* Modeling methane emission from rice paddies with various agricultural practices. *Journal of Geophysical Research: Atmospheres*. 2004; **109**(D8).

28. Gilbert M, Cinardi G, Da Re D *et al.* Global distribution data for cattle, buffaloes, horses, sheep, goats, pigs, chickens and ducks in 2015 (5 minutes of arc). Harvard Dataverse. 2022. Available at https://doi.org/10.7910/DVN/SXHLF3

29. Gilbert M, Nicolas G, Cinardi G *et al.* Global distribution data for cattle, buffaloes, horses, sheep, goats, pigs, chickens and ducks in 2010. 2018. Available at https://doi.org/10.1038/sdata.2018.227

30. Song C, Fan C, Zhu J *et al.* A comprehensive geospatial database of nearly 100000 reservoirs in China. *Earth System Science Data*. 2022; **14**(9): 4017-4034.

31. Zhang G, Yao T, Chen W *et al.* Regional differences of lake evolution across China during 1960s–2015 and its natural and anthropogenic causes. *Remote Sensing of Environment*. 2019; **221**: 386-404.

32. Luan S *et al*. The national assessment of GHG emissions from lakes and reservoirs. *Under review in this issue.*

33. Lou Z *et al*. High-resolution mapping of CH4 and N2O emissions from energy, industry and the waste sectors in China. *Under review in this issue.*

34. Wang X, Gao Y, Wang K *et al.* The greenhouse gas budget for China's terrestrial ecosystems. *National Science Review*. 2023; **10**(12).

35. Jiang F, Chen JM, Zhou L *et al.* A comprehensive estimate of recent carbon sinks in China using both top-down and bottom-up approaches. *Scientific Reports*. 2016; **6**: 22130.

36. Wang Y, Wang X, Wang K *et al.* The size of the land carbon sink in China. *Nature*. 2022; **603**(7901): E7-E9.

37. Van Der Werf GR, Randerson JT, Giglio L *et al.* Global fire emissions estimates during 1997–2016. *Earth System Science Data*. 2017; **9**(2): 697-720.

38. Yang S, Chang BX, Warner MJ *et al.* Global reconstruction reduces the uncertainty of oceanic nitrous oxide emissions and reveals a vigorous seasonal cycle. *Proceedings of the National Academy of Sciences*. 2020; **117**(22): 11954-11960.

39. Shen L, Zavala-Araiza D, Gautam R *et al.* Unravelling a large methane emission discrepancy in Mexico using satellite observations. *Remote Sensing of Environment*. 2021; **260**: 112461.

40. Turner AJ, Jacob DJ. Balancing aggregation and smoothing errors in inverse models. *Atmospheric Chemistry and Physics*. 2015; **15**(12): 7039-7048.

41. Shen L, Gautam R, Omara M *et al.* Satellite quantification of oil and natural gas methane emissions in the US and Canada including contributions from individual basins. *Atmospheric Chemistry and Physics*. 2022; **22**(17): 11203-11215.

42. Zhang H, Lauerwald R, Regnier P *et al.* Estimating the lateral transfer of organic carbon through the European river network using a land surface model. *Earth System Dynamics*. 2022; **13**(3): 1119-1144.

43. Zhang H, Lauerwald R, Ciais P *et al.* Global changes alter the amount and composition of land carbon deliveries to European rivers and seas. *Communications Earth & Environment*. 2022; **3**(1): 245.

44. Liu D, Bai Y, He XQ *et al.* Changes in riverine organic carbon input to the ocean from mainland China over the past 60 years. *Environ Int*. 2020; **134**: 105258.

45. Wang XY, Quine TA, Zhang HC *et al.* Redistribution of Soil Organic Carbon Induced by Soil Erosion in the Nine River Basins of China. *Journal of Geophysical Research-Biogeosciences*. 2019; **124**(4): 1018-1031.

46. Xia J *et al*. China Carbon 1.0: a multiple carbon cycle model intercomparison project. *Under review in this issue.*

47. Saunois M, Martinez A, Poulter B *et al.* Global Methane Budget 2000-2020. *Earth System Science Data Discussions*. 2024; **2024**: 1-147.

48. U.S. Department of Agriculture ARS. USDA Food and Nutrient Database for Dietary Studies 2019-2020. Food Surveys Research Group. 2022. Available at http://www.ars.usda.gov/nea/bhnrc/fsrg

49. Ciais P, Bousquet P, Freibauer A *et al.* Horizontal displacement of carbon associated with agriculture and its impacts on atmospheric CO2. *Global Biogeochemical Cycles*. 2007; **21**(2).

50. Baes ICF, Sharp RD, Sjoreen AL *et al.* Review and analysis of parameters for assessing transport of environmentally released radionuclides through agriculture. United States 1984. Available at https://www.osti.gov/biblio/6355677
